# Telomere-to-Telomere Genome Assemblies of *Coprinellus xanthothrix* and *Coprinellus saccharinus* Reveal Chromosome-Scale Genome Architecture and Lineage-Specific Evolutionary Dynamics

**DOI:** 10.3390/jof12090678

**Published:** 2026-09-10

**Authors:** Minghan Yang, Wenyan Huo, Haoxuan Li, Junzhi Li, Xuelian He, Lu Dai, Peng Qi, Yu Liu, Liguang Zhang, Ting Qiao, Guanglin Li

**Affiliations:** 1 School of Life Science and Technology, Harbin Institute of Technology, Harbin 150001, China; yummyhan0413@gmail.com; 2 Fungal Research Center, Shaanxi Provincial Institute of Microbiology, Xi’an 710043, China; huowenyanabace@gmail.com (W.H.); m15529200671@163.com (H.L.); xianjunzhi@163.com (J.L.); xuelhe@163.com (X.H.); dailutk@126.com (L.D.); qipeng_325@163.com (P.Q.); ly137261323@126.com (Y.L.); liguang_zhang@163.com (L.Z.); syjaqt@163.com (T.Q.); 3 College of Life Science, Shaanxi Normal University, Xi’an 710062, China

**Keywords:** *Coprinellus xanthothrix*, *Coprinellus saccharinus*, telomere-to-telomere genome, comparative genomics, chromosome architecture, retroelements, gene family evolution, synteny, carbohydrate-active enzymes, whole-genome duplication

## Abstract

*Coprinellus* comprises mushroom-forming saprotrophic fungi that colonize decomposing plant-derived substrates, yet chromosome-complete genomic resources for this genus remain limited. To resolve chromosome architecture and lineage-specific evolution in this fungal group, we generated chromosome-complete assemblies of *Coprinellus xanthothrix* and *C. saccharinus* by integrating Oxford Nanopore Technologies (ONT) long reads, DNA nanoball sequencing (DNBSEQ) short reads, Hi-C, and RNA-seq data. Each genome comprised 13 gapless chromosomes with all 26 telomeres recovered; assembly sizes were 46.92 and 54.81 Mb, with BUSCO completeness of 99.20% and 99.10%, respectively. The larger *C. saccharinus* genome was primarily associated with greater retroelement content, whereas functional annotation profiles and CAZyme repertoires were broadly comparable between species. Phylogenomic analyses grouped *C. xanthothrix* with *C. radians* and *C. saccharinus* with *C. micaceus*, with estimated divergence times of 54.4 and 32.8 Ma, respectively. Both divergence events occurred within the Paleogene. Gene family analyses identified lineage-specific expansions enriched in nucleotide metabolism, DNA replication and repair, glutathione metabolism, redox regulation, endocytosis, cytoskeletal organization, and cell-cycle processes. Synteny and Ks analyses further revealed chromosome-level rearrangements and lineage-specific small-scale duplication but no strong evidence of recent whole-genome duplication. Together, the temporal placement of these divergences and the associated genomic patterns raise a testable hypothesis that long-term climatic and vegetation reorganization, together with changes in plant-derived substrates and microhabitats, may have contributed to lineage establishment and ecological differentiation. However, because direct paleoecological evidence and functional validation are currently lacking, this proposed relationship should not be interpreted as causal. These chromosome-complete assemblies provide important resources for comparative genomics in *Coprinellus* and future experimental studies of saprotrophic adaptation.

## 1. Introduction

Fungal genomes contain biologically important repeats, chromosome-end features, and structural variants that can be missed or misassembled in draft assemblies. Complete and chromosome-end-resolved genome assemblies are therefore becoming foundational resources for fungal comparative genomics. Although draft assemblies have supported many analyses of gene content, orthology, and species relationships, fragmented contigs can obscure chromosome termini, subtelomeric repeats, repeat-rich intervals, local duplication structure, and long-range syntenic relationships. The telomere-to-telomere (T2T) assembly framework has consequently become an important direction in genomics, particularly after rapid improvements in long-read sequencing, polishing, and chromosome-conformation scaffolding [1,2].

Recent fungal T2T or near-T2T studies show why complete assemblies matter for mycology. A telomere-to-telomere genome of the model mould pathogen *Aspergillus fumigatus* improved resolution of chromosome architecture and repetitive regions [3]. Complete chromosome-end assemblies of *Agaricus bisporus* revealed polymorphic chromosome ends in the cultivated button mushroom [4]. In macrofungi, the T2T assembly of *Ganoderma leucocontextum* supported centromere-associated repeat characterization [5], and a haplotype-resolved T2T assembly of the dikaryotic pathogen *Rhizoctonia cerealis* showed how complete assembly can clarify haplotype structure and genome organization [6]. These studies collectively indicate that complete assemblies are not simply longer versions of draft genomes; they provide a more reliable coordinate system for studying repeats, telomeres, synteny, and lineage-specific genome evolution.

*Coprinellus* is a mushroom-forming genus in *Psathyrellaceae*. The modern circumscription of coprinoid fungi was reshaped when *Coprinus* sensu lato was divided into *Coprinus* sensu stricto, *Coprinellus*, *Coprinopsis*, and *Parasola*, with *Coprinellus* placed in *Psathyrellaceae* [7]. Subsequent multigene phylogenetic work demonstrated that species delimitation within *Coprinellus*, especially among haired species, can be complex and benefits from molecular data [8]. Related coprinoid fungi have also been important for fungal genomics: the assembled chromosomes of *Coprinopsis cinerea* provided an early model for studying multicellular development and chromosome biology in mushroom-forming fungi [9]. More broadly, *Coprinellus* species are saprotrophic fungi that colonize decomposing organic substrates such as wood, leaves, grass, and dung, linking their genome evolution to plant-derived carbon turnover and microhabitat specialization [10].

Despite this biological and phylogenetic relevance, chromosome-complete resources for *Coprinellus* remain limited. Existing comparative datasets include several *Coprinellus* and related fungal genomes, but most available assemblies do not resolve both telomeres of every chromosome. This limitation is important because chromosome-end regions and repetitive sequences can contribute to genome-size variation, structural rearrangement, and gene family evolution. For saprotrophic mushrooms, complete genomes also provide a better framework for interpreting carbohydrate-active enzyme (CAZyme) repertoires, gene family expansions, and potential links between genome architecture and ecological adaptation.

In the present study, we generated T2T genome assemblies for *Coprinellus xanthothrix* (CXA) and *Coprinellus saccharinus* (CSA). These two species occupy different positions within the local *Coprinellus* comparative framework: *C. xanthothrix* is closely related to *C. radians*, whereas *C. saccharinus* is closely related to *C. micaceus*. This arrangement allows the two new assemblies to anchor separate lineage comparisons while being processed under the same assembly, annotation, and evolutionary-analysis framework. We combined ONT long reads, DNBSEQ short reads, Hi-C data, and RNA-seq data to assemble and validate the genomes, then annotated repeats, genes, functional categories, and CAZymes. We further inferred orthogroups across 11 fungal genomes, reconstructed a dated phylogeny, estimated gene family expansion and contraction, tested GO and KEGG enrichment among expanded gene families, and examined chromosome-scale synteny and synonymous substitution rate (Ks) distributions.

The goals of this work were threefold. First, we aimed to produce high-quality telomere-capped chromosome assemblies for *C. xanthothrix* and *C. saccharinus*. Second, we sought to compare their genome size, repeat composition, gene content, CAZyme repertoire, and chromosome-level organization using the same analytical framework. Third, we evaluated lineage-specific gene family dynamics and considered whether the Paleogene divergence of *C. xanthothrix* and *C. saccharinus* from close relatives may have coincided with broader climatic warming, forest ecological expansion, and ecological niche restructuring. The resulting assemblies provide reference genomes for *Coprinellus* comparative genomics and a data-supported basis for future functional studies of saprotrophic mushroom adaptation.

## 2. Materials and Methods

### 2.1. Fungal Strains and Culture Conditions

*Coprinellus xanthothrix* strain 005 and *C. saccharinus* strain 020 were collected in the Qinling Mountains of Shaanxi Province, China, and cultured to yield fresh mycelial biomass for genomic DNA and total RNA extraction. The fungal cultures were incubated in potato dextrose broth (Beijing Aoboxing Bio-Tech Co., Ltd., Beijing, China) within a temperature-controlled orbital shaker (Shanghai Zhicheng Analytical Instrument Manufacturing Co., Ltd., Shanghai, China) at 25 °C for 5–7 days. Mycelia were harvested via filtration, snap-frozen in liquid nitrogen immediately, and preserved at −80 °C prior to nucleic acid extraction. Species identification was verified based on internal transcribed spacer (ITS) sequences.

### 2.2. Nucleic Acid Extraction and Quality Assessment

High-molecular-weight genomic DNA was extracted from frozen mycelial material using the NucleoBond^®^ HMW DNA Kit (MACHEREY-NAGEL GmbH & Co. KG, Düren, Germany), while total RNA was extracted using TRIzol^TM^ Reagent (Invitrogen, Thermo Fisher Scientific, Carlsbad, CA, USA). Nucleic acid purity was assessed spectrophotometrically with a NanoDrop™ instrument (Thermo Fisher Scientific, Waltham, MA, USA), and nucleic acid concentration was quantified using a Qubit^®^ 3.0 fluorometer (Thermo Fisher Scientific). The integrity of DNA and RNA was further evaluated via 1% agarose gel electrophoresis (Beijing Liuyi Biotechnology Co., Ltd., Beijing, China) to confirm that all samples satisfied the quality standards required for library preparation. Qualified samples that passed all quality assessment criteria were subjected to Oxford Nanopore Technologies (ONT) long-read sequencing, DNA nanoball sequencing (DNBSEQ) short-read whole-genome sequencing, Hi-C library construction, and RNA-seq library construction. All the above quality-control procedures adopted the general analytical framework, guaranteeing that sequencing libraries were generated from intact nucleic acids with minimal contamination.

### 2.3. Library Construction and Sequencing Platform Integration

Four complementary sets of sequencing data were generated for each species. Oxford Nanopore Technologies (ONT) sequencing was applied to generate ultra-long reads capable of spanning repetitive regions, which served as the primary dataset for de novo contig assembly and telomere extension. High-molecular-weight genomic DNA was subjected to end repair and enzymatic single-strand conversion prior to adapter ligation. Nanopore libraries were constructed and sequenced on a PromethION instrument (Oxford Nanopore Technologies, Oxford, UK) with R10.4.1 flow cells (Oxford Nanopore Technologies, Oxford, UK). Raw electrical signals were base-called via Dorado (v0.9.0, https://github.com/nanoporetech/dorado, accessed on 6 March 2026) to yield high-quality ultra-long ONT reads [11].

DNBSEQ short-read sequencing was performed to support genome survey analysis and single-nucleotide-level genome polishing on an MGI DNBSEQ high-throughput sequencer (MGI Tech Co., Ltd., Shenzhen, China). Genomic DNA was fragmented physically or enzymatically, followed by end repair, dA-tailing and adapter ligation. Target-size DNA fragments were purified with magnetic beads (MGI Tech Co., Ltd., Shenzhen, China) and denatured into single-stranded circles for rolling circle amplification to produce DNA nanoballs (DNBs). Libraries were sequenced in paired-end 150 bp (PE150) mode [12].

Hi-C sequencing data were used to construct chromosome-scale scaffolds, validate chromosomal architecture, and provide contact information for the final genome assemblies. Briefly, fresh mycelial cells were cross-linked with formaldehyde to preserve native chromatin conformation and digested with DpnII restriction endonuclease (New England Biolabs, Ipswich, MA, USA). Biotin-labelled nucleotides were incorporated during proximity ligation at cleavage sites. Following cross-link reversal and DNA purification, DNA was sheared to fragments ranging from 300 to 700 bp. Biotin-containing ligation products were enriched using streptavidin magnetic beads (Thermo Fisher Scientific, Waltham, MA, USA), and the resulting Hi-C libraries were sequenced in PE150 mode on the DNBSEQ-T7 platform (MGI Tech Co., Ltd., Shenzhen, China) [13].

RNA-seq transcriptome data were generated to supply transcript evidence for gene prediction and improve genome functional annotation. Poly(A)+ mRNA was enriched from total RNA using oligo-dT magnetic beads (Thermo Fisher Scientific, Waltham, MA, USA). After chemical denaturation, mRNA was reverse-transcribed into double-stranded cDNA with random hexamers (Thermo Fisher Scientific, Waltham, MA, USA). The cDNA was processed through end repair, 3′ dA-tailing, adapter ligation and PCR amplification to construct single-stranded circular DNA libraries, which were subsequently sequenced in PE150 mode on the DNBSEQ platform (MGI Tech Co., Ltd., Shenzhen, China). The sequencing depths and datasets obtained from the four sequencing strategies are summarised in Table S10 [14].

### 2.4. Genome Survey and Preliminary Characterization

Prior to the final genome assembly, k-mer frequency distribution analysis was performed to characterize the preliminary genomic features of the strains. Quality-filtered clean short reads were subjected to k-mer analysis with a k-value of 21 using Jellyfish (v2.2.10) (The Center for Computational Biology, Johns Hopkins University, Baltimore, MD, USA) [15], and the resulting k-mer frequency spectra were fitted via GenomeScope 2.0 (https://github.com/schatzlab/genomescope, accessed on 6 March 2026) [16] to estimate genome size, heterozygosity level, and genomic repeat content. The obtained genomic survey results not only revealed the overall size and complexity of the genome but also guided the parameter optimization for subsequent genome assembly tools, while providing a reliable baseline for evaluating the size and completeness of the final assembled genome. Adapter trimming and strict quality filtering of short reads were conducted in advance to ensure the accuracy of downstream k-mer analysis and genome assembly [17,18].

### 2.5. Data Quality Control and T2T Assembly Pipeline

Raw sequencing reads from multiple platforms were first subjected to strict quality filtering and trimming to eliminate adapter sequences, low-quality bases, ultra-short fragments, and technical sequencing artifacts. Specifically, DNBSEQ short reads were processed and trimmed using fastp (v0.23.2) (https://github.com/OpenGene/fastp, accessed on 6 March 2026) [17], Hi-C paired-end reads were cleaned with Trim Galore (v0.6.7) (https://github.com/FelixKrueger/TrimGalore, accessed on 6 March 2026) [18], and ultra-long ONT reads were filtered via Filtlong (v0.2.1) (https://github.com/rrwick/Filtlong, accessed on 6 March 2026) [19] to retain high-quality valid sequences for subsequent genome assembly. The cleaned ONT ultra-long reads were corrected and assembled de novo using NECAT (https://github.com/xiaochuanle/NECAT, accessed on 7 March 2026) [20] to generate the initial draft contigs. To improve assembly accuracy, iterative polishing was performed: the draft contigs were firstly polished for multiple rounds with Racon (v1.4.3) (https://github.com/isovic/racon, accessed on 7 March 2026) [21], with ONT reads realigned to the updated contig references using minimap2 (https://github.com/lh3/minimap2, accessed on 7 March 2026) [22] in each iteration. For the elimination of residual single-base and small structural errors, high-quality short reads were mapped to the polished contigs using BWA-MEM (https://github.com/lh3/bwa, accessed on 7 March 2026) [23], followed by sorting and indexing of alignment files via SAMtools (v1.22.1) (https://github.com/samtools/samtools, accessed on 7 March 2026) [24], and two additional rounds of base-level refinement were conducted using Pilon (v1.24) (https://github.com/broadinstitute/pilon, accessed on 7 March 2026) [25].

For chromosome-scale scaffolding, Juicer (v1.6) (https://github.com/aidenlab/juicer, accessed on 7 March 2026) [26] and 3D-DNA (https://github.com/aidenlab/3d-dna, accessed on 7 March 2026) [27] were applied to process Hi-C alignment data and construct chromosomal scaffolds, with Hi-C contact maps generated at a 100 kb resolution for structural evaluation. Preliminary assembly errors and structural abnormalities were manually inspected and corrected using Juicebox Assembly Tools (https://github.com/aidenlab/Juicebox, accessed on 7 March 2026) [28]. The manually curated assembly was further processed to generate standardized chromosome-level FASTA sequences utilizing the assembly2agp and agp2fasta modules of CPhasing (v0.2.1, https://github.com/wangyibin/CPhasing, accessed on 7 March 2026) to optimize chromosomal grouping and structural ordering.

To achieve complete telomere-capped gapless genomes, FungalTeloExtender (v1.0.3, https://github.com/huowenyanabace/FungalTeloExtender, accessed on 8 March 2026) was employed with default parameters for systematic telomere motif identification and sequence extension.

The identical assembly pipeline with consistent software versions and parameter settings was uniformly applied for the de novo genome assembly of *C. xanthothrix* and *C. saccharinus*. Comprehensive assembly statistics and quality metrics of the two target species are summarized in Table 1. All final assemblies were gapless telomere-to-telomere (T2T) genomes with all chromosomal ends fully capped by the telomere motif AACCCT. The two assemblies achieved BUSCO (https://busco.ezlab.org, accessed on 9 March 2026) completeness scores of 99.20% (*C. xanthothrix*) and 99.10% (*C. saccharinus*) based on the fungi_odb10 database, with respective QV values of 38.36 and 39.26. The uniformly assembled high-quality genomes provide reliable and comparable genomic resources for subsequent analyses of orthologous relationships, CAZyme annotation, synteny comparison, and gene family evolution.

### 2.6. Assembly Quality Validation

Assembly quality was evaluated using multiple complementary metrics. Contiguity and chromosome number were assessed from final scaffold statistics, including genome size, chromosome number, largest and smallest chromosome length, N50, N90, L50, and L90. Assembly contiguity was summarized using QUAST-style metrics (http://quast.sourceforge.net, accessed on 9 March 2026) [29], base-level accuracy was estimated using consensus quality value (QV) (Merqury, https://github.com/marbl/merqury, accessed on 9 March 2026) [30], and gene-space completeness was evaluated using BUSCO with an appropriate fungal lineage dataset [31]. Telomere completeness was assessed by searching chromosome termini for the canonical fungal telomeric repeat motifs 5-prime (AACCCT)n and 3-prime (AGGGTT)n. Hi-C contact maps were visualized and inspected using Hi-C contact-map analysis approaches (HiCExplorer, https://hicexplorer.readthedocs.io, accessed on 9 March 2026) [32] to assess chromosome-scale structural consistency.

### 2.7. Repetitive Element Annotation

Repetitive elements were annotated using a combined strategy that incorporated de novo repeat prediction and homology-based repeat searches. Repeat classes included retroelements, DNA transposons, rolling-circle elements, small RNA-related repeats, simple repeats, and low-complexity regions. RepeatModeler2 (v2.0.1) (https://github.com/Dfam-consortium/RepeatModeler, accessed on 10 March 2026), RepeatMasker (v4.2.2) (https://www.repeatmasker.org, accessed on 10 March 2026), and Repbase-supported searches (https://www.girinst.org/repbase, accessed on 10 March 2026) were used to mask repeat sequences and summarize repeat composition [33,34,35]. Class-level repeat landscapes are reported in Table 2, and detailed family-level repeat compositions for CXA and CSA are provided in Tables S7 and S8, respectively.

### 2.8. Gene Prediction, Functional Annotation, and CAZyme Detection

Protein-coding genes were predicted using an integrated annotation strategy combining ab initio prediction with transcriptomic and homology-based evidence. RNA-seq reads were aligned to the genome using HISAT2 (https://daehwankimlab.github.io/hisat2/, accessed on 10 March 2026) [36], and the resulting alignments were used as empirical evidence for an initial round of gene prediction with BRAKER v2.1.6 (https://github.com/Gaius-Augustus/BRAKER, accessed on 10 March 2026) [37]. Final gene prediction and evidence integration were performed using MAKER v3.1.3 [38], incorporating de novo assembled transcripts generated with rnaSPAdes v3.15.5 [39], transcripts from closely related species, homologous proteins retrieved from NCBI databases, and Augustus training parameters (http://bioinf.uni-greifswald.de/augustus, accessed on 10 March 2026) generated during the BRAKER analysis [40]. These predicted proteins were used for functional annotation with eggNOG-mapper v2.1.12 (http://eggnog-mapper.embl.de, accessed on 10 March 2026) [41], which assigned GO, KEGG, and COG/KOG terms through homology-based searches against complete reference databases (https://www.ncbi.nlm.nih.gov/COG, https://www.genome.jp/kegg, accessed on 10 March 2026).

CAZymes were identified using run_dbcan v2.0.11 from the dbCAN2 suite (https://bcb.unl.edu/dbCAN2, accessed on 11 March 2026) [42] through three complementary approaches: HMMER searches against dbCAN HMM profiles (http://hmmer.org, accessed on 11 March 2026) (E-value < 1 × 10^−5^ and coverage > 0.35) [43], DIAMOND searches against CAZyDB (https://github.com/bbuchfink/diamond, accessed on 11 March 2026) (identity > 30% and coverage > 0.35) [44], and Hotpep peptide-based searches (https://bcb.unl.edu/hotpep, accessed on 11 March 2026) (frequency ≥ 2.6 and hits ≥ 6) [45]. To improve annotation reliability and minimize false-positive assignments, only genes supported by at least two of the three methods were retained in the final CAZyme catalogue. The identified CAZymes were classified as Glycoside Hydrolases (GHs), Glycosyl Transferases (GTs), Polysaccharide Lyases (PLs), Carbohydrate Esterases (CEs), Auxiliary Activities (AAs), and Carbohydrate-binding Modules (CBMs). Gene annotation statistics are summarized in Table S11, whereas class- and family-level CAZyme distributions are presented in Tables S2 and S3, respectively.

### 2.9. Genome Visualization and Circos Plot Generation

Genome-wide feature landscapes were visualized for both species. Chromosome ideograms, GC content, gene density, repeat density, and intragenomic synteny were summarized in Circos-style plots (Circos v0.69-8, http://circos.ca, accessed on 9 March 2026) [46]. The window-based metrics for GC content, gene density, and repeat coverage were estimated using Bedtools (v2.30.0) (https://bedtools.readthedocs.io, accessed on 9 March 2026) [47] and seqtk (https://github.com/lh3/seqtk, accessed on 9 March 2026) with properly sized sliding windows to balance resolution and visualization clarity. Hi-C contact matrices were used to visualize genome-wide and per-chromosome interaction patterns. These visualizations were used to support interpretation of chromosome integrity, genome organization, and feature distribution across the two T2T assemblies.

### 2.10. Comparative Genomics Framework

Comparative genomics included *C. xanthothrix*, *C. saccharinus*, and nine related fungal genomes: *C. domesticus*, *C. radians*, *C. disseminatus*, *C. micaceus*, *Coprinopsis cinerea*, *Coprinopsis marcescibilis*, *Psathyrella aberdarensis*, *Agaricus bisporus*, and *Saccharomyces cerevisiae* as the outgroup (Table S1). Orthology inference across the 11 fungal genomes was conducted with OrthoFinder v2.5.4 (https://github.com/davidemms/OrthoFinder, accessed on 9 March 2026) [48]. DIAMOND [44] was employed for rapid sequence-similarity searches, while multiple sequence alignments were generated using MAFFT (https://mafft.cbrc.jp/alignment/software, accessed on 9 March 2026) [49]. Phylogenetic relationships among the sampled species were reconstructed using two complementary strategies: a maximum-likelihood analysis of the concatenated orthologue alignment with raxmlHPC-PTHREADS (https://github.com/stamatak/standard-RAxML, accessed on 9 March 2026) [50] and a multispecies coalescent analysis with ASTRAL v5.7.8 (https://github.com/smirarab/ASTRAL, accessed on 9 March 2026) [51]. Assembly accessions for all comparison genomes are provided in Table S1; the two focal assemblies are deposited under GCA_059941265.1 and GCA_059941275.1.

The divergence times of major evolutionary lineages were inferred with MCMCTree implemented in PAML v4.9 [52]. The split between *S. cerevisiae* and *Psathyrellaceae* was selected as the principal temporal constraint, with soft lower and upper bounds of 583 and 749 million years ago, respectively, based on the TimeTree database (http://www.timetree.org, accessed on 9 March 2026) [53]. Markov chain Monte Carlo sampling was performed for 2,000,000 generations, of which the first 500,000 were discarded as burn-in. Adequate convergence was supported by effective sample sizes exceeding 200 for all estimated parameters. The posterior median divergence ages and associated 95% highest posterior density intervals for the principal nodes are provided in Table S6.

### 2.11. Gene Family Evolution and Functional Enrichment

Gene family expansion and contraction were estimated using CAFE (https://github.com/hahnlab/CAFE, accessed on 9 March 2026) [54] based on the dated species tree and orthogroup counts. Terminal and internal branches were examined for expanded and contracted gene families, genes gained and lost, and rapidly evolving families. Expanded gene families in *C. xanthothrix* and *C. saccharinus* were subjected to GO and KEGG enrichment analyses using enrichment-analysis workflows such as clusterProfiler (https://bioconductor.org/packages/clusterProfiler, accessed on 9 March 2026) [55]. KEGG pathway identifiers shown in enrichment figures were interpreted using the pathway-name mapping in Table S9.

### 2.12. Synteny Analysis and Ks-Based Duplication Assessment

Chromosome-scale synteny was evaluated among selected *Coprinellus* species using pairwise genome alignment and syntenic block detection (JCVI v1.3.6, https://github.com/tanghaibao/jcvi, accessed on 9 March 2026) [47,56]. Macrosynteny was visualized for *C. xanthothrix* with *C. domesticus* and *C. radians*, and for *C. saccharinus* with *C. disseminatus*. Dot-plot analyses were generated for *C. xanthothrix* versus *C. domesticus*, *C. xanthothrix* versus C. radians, and *C. saccharinus* versus *C. disseminatus*. Synonymous substitution rate (Ks) distributions were calculated for paralogous gene pairs within *C. xanthothrix* and *C. saccharinus* and for orthologous gene pairs between *C. xanthothrix* and *C. domesticus*, *C. xanthothrix* and *C. radians*, *C. saccharinus* and *C. disseminatus*, and *C. saccharinus* and *C. micaceus* (https://github.com/arzwa/wgd, accessed on 9 March 2026) [57].

### 2.13. Data Visualization and Statistical Software

Final plots were prepared from the processed assembly, annotation, orthology, enrichment, synteny, and Ks outputs. Statistical summaries were reported directly from the corresponding tables and figure source files. Unless otherwise specified, descriptive comparisons were based on observed counts, proportions, assembly metrics, enrichment outputs, and divergence-time estimates generated by the pipelines described above. Custom scripts written in Python v3.11 and R v4.5 were used to process the data and generate the figures. The complete source code supporting these analyses and visualizations is publicly available in the GitHub repository (https://github.com/huowenyanabace/T2Tfungi_custom_scripts.git, accessed on 9 March 2026).

## 3. Results

### 3.1. Sequencing Data Generation and Coverage Statistics

Multi-platform sequencing generated sufficient data for chromosome-complete assembly and annotation of both *Coprinellus* species (Table S10). For *C. xanthothrix*, DNBSEQ short-read sequencing produced 15.22 Gb of raw data and 15.12 Gb of clean data, corresponding to 101.48 million raw reads, 101.47 million clean reads, and approximately 324× coverage. ONT long-read sequencing generated 10.76 Gb raw data and 10.38 Gb clean data, with 1.77 million raw reads, 1.62 million clean reads, and approximately 228× coverage. Hi-C sequencing generated 10.85 Gb raw data and 10.84 Gb clean data, corresponding to 36.18 million raw and clean reads and approximately 231× coverage. RNA-seq produced 5.49 Gb of raw data and 0.11 million raw reads for transcript-supported gene annotation.

For *C. saccharinus*, short-read sequencing generated 10.28 Gb raw data and 10.03 Gb clean data, corresponding to 68.52 million raw reads, 67.92 million clean reads, and approximately 187× coverage (Table S10). ONT sequencing generated 12.57 Gb raw data and 11.96 Gb clean data, with 0.54 million raw reads, 0.50 million clean reads, and approximately 229× coverage. Hi-C sequencing produced 19.11 Gb raw data and 19.09 Gb clean data, corresponding to 63.69 million raw and clean reads and approximately 349× coverage. RNA-seq generated 9.12 Gb of raw data and 0.19 million raw reads. These datasets provided complementary long-read, short-read, chromosome-conformation, and transcriptomic evidence for the two assemblies.

### 3.2. T2T Assembly Quality, Structural Validation, and Telomere Integrity

**Figure 1:**
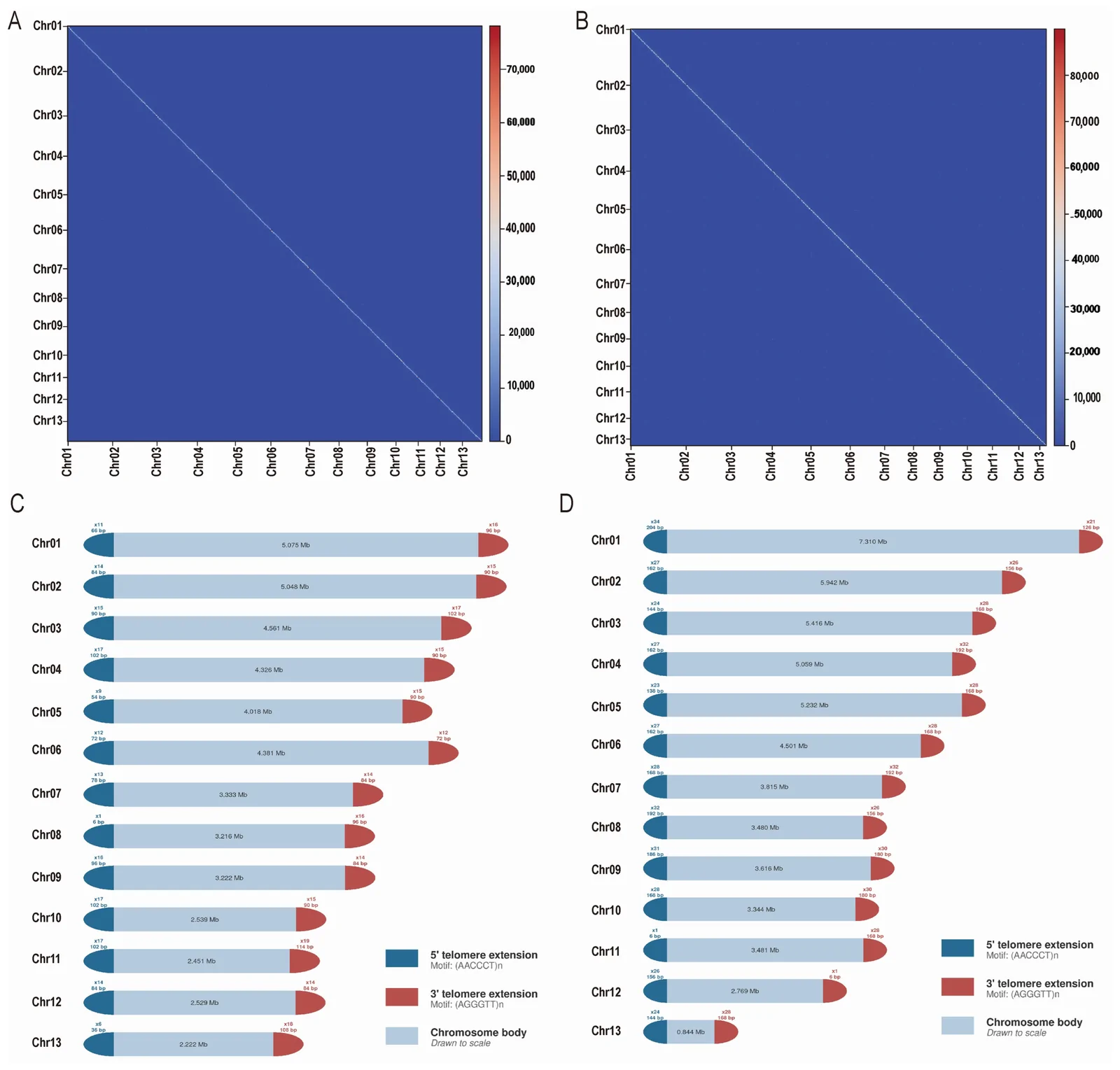
Telomere-to-telomere genome assemblies of *C. xanthothrix* and *C. saccharinus*. (**A**,**B**) Genome-wide Hi-C contact maps at 100 kb resolution for *C. xanthothrix* (**A**) and *C. saccharinus* (**B**), supporting the chromosome-scale continuity and structural integrity of the assemblies. (**C**,**D**) Chromosome-level ideograms of *C. xanthothrix* (**C**) and *C. saccharinus* (**D**), showing chromosome bodies drawn to scale and terminal telomere extensions with 5′ (AACCCT)n and 3′ (AGGGTT)n motifs.

Both *C. xanthothrix* and *C. saccharinus* were assembled into telomere-to-telomere (T2T) chromosome-scale genomes (Table 1; Figure 1). The *C. xanthothrix* genome was 46.92 Mb and consisted of 13 gapless chromosomes. Its largest chromosome was Chr01 at 5.08 Mb and its smallest chromosome was Chr13 at 2.22 Mb. The assembly had a GC content of 53.72%, N50 of 4,017,987 bp, N90 of 2,528,967 bp, L50 of 6, L90 of 11, QV of 38.36, and BUSCO completeness of 99.20%. The *C. saccharinus* assembly was larger, at 54.81 Mb, and also consisted of 13 gapless chromosomes. Its largest chromosome was Chr01 at 7.31 Mb and its smallest chromosome was Chr13 at 0.84 Mb. The *C. saccharinus* assembly had a GC content of 53.79%, N50 of 5,059,299 bp, N90 of 3,343,676 bp, L50 of 5, L90 of 11, QV of 39.26, and BUSCO completeness of 99.10%.

Hi-C contact maps supported the chromosome-scale organization of both assemblies (Figure 1A,B). The interaction matrices showed strong intrachromosomal contact patterns for each chromosome, consistent with the 13-chromosome assembly structure. Per-chromosome Hi-C heatmaps further supported the structural integrity of all 13 *C. xanthothrix* chromosomes (Figure S1) and all 13 *C. saccharinus* chromosomes (Figure S2). Chromosome ideograms showed continuous chromosome models with terminal telomere extensions at both ends (Figure 1C,D). Telomere analysis identified the expected 5-prime (AACCCT)n and 3-prime (AGGGTT)n motifs and recovered 26 of 26 telomere-capped chromosome ends in each species (Table 1; Table S5). Terminal extension lengths varied among chromosomes and termini; for example, *C. xanthothrix* Chr01 had a 155,584 bp 3-prime extension and a 34,865 bp 5-prime extension, whereas *C. saccharinus* Chr01 had a 153,876 bp 3-prime extension and a 921 bp 5-prime extension (Table S5).

### 3.3. Chromosome-Level Genomic Landscapes

**Figure 2:**
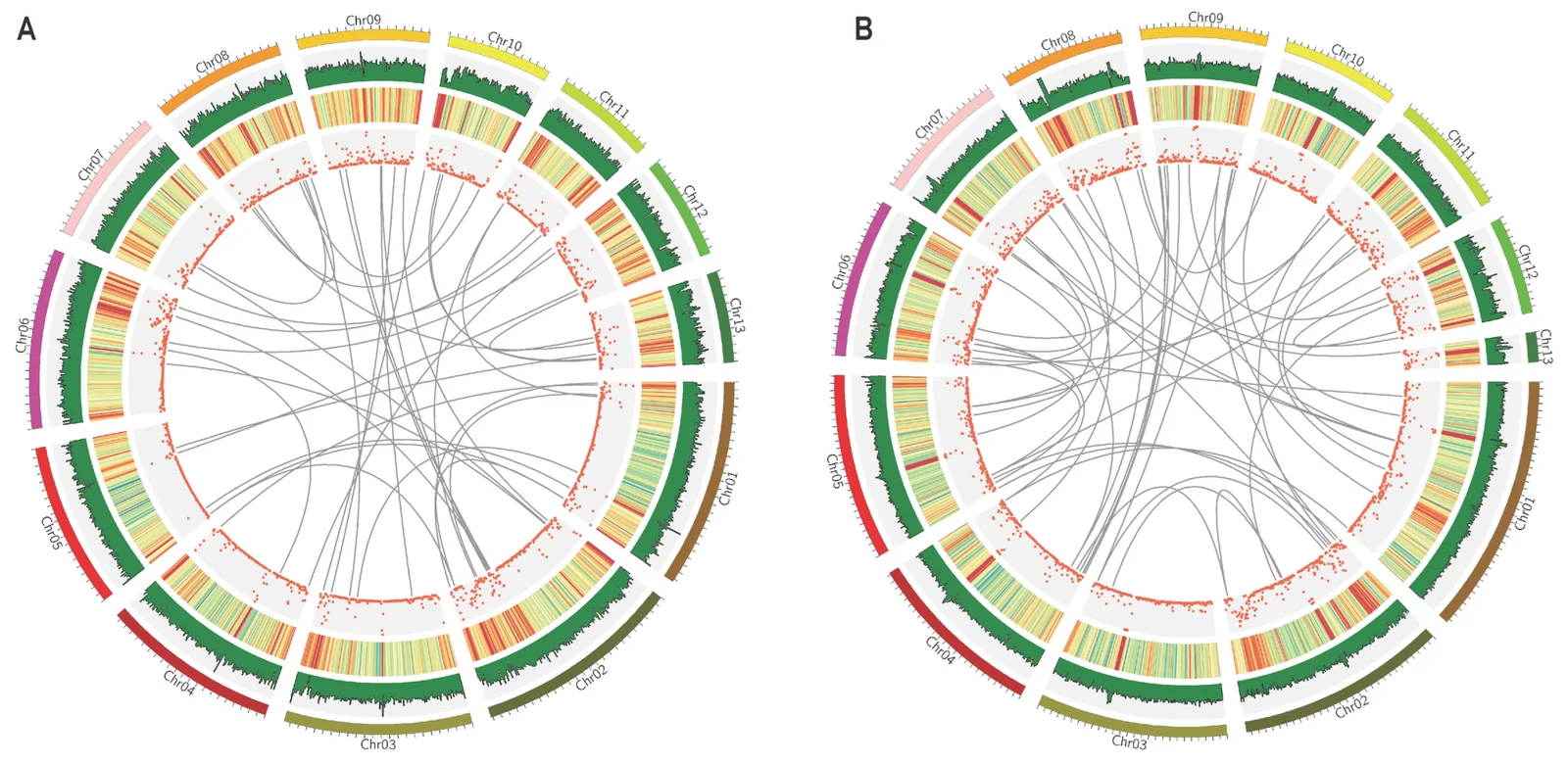
Circos visualization of chromosome organization and genomic feature landscapes in *C. xanthothrix* and *C. saccharinus*. (**A**) *C. xanthothrix* and (**B**) *C. saccharinus.* Tracks from outside to inside represent chromosome ideograms, GC content, gene density, repeat density, and intragenomic synteny ribbons.

Circos plots summarized genome-wide feature distributions across the two T2T assemblies (Figure 2). The outer chromosome tracks showed the 13 assembled chromosomes of *C. xanthothrix* and *C. saccharinus*, while inner tracks displayed GC content, gene density, repeat density, and intragenomic synteny ribbons. The visualization showed that both genomes have chromosome-wide variation in gene and repeat density, with repeat-enriched regions contributing to local heterogeneity. Intragenomic synteny links indicated duplicated or homologous segments within each genome. Together with the Hi-C evidence in Figure 1 and Figures S1 and S2, the Circos plots support the interpretation that both assemblies provide complete chromosome frameworks suitable for repeat, gene-density, and synteny analyses.

### 3.4. Repetitive Element Landscapes

Repeat annotation revealed a larger repeat fraction in *C. saccharinus* than in *C. xanthothrix* (Table 2 and Tables S7 and S8). In *C. xanthothrix*, interspersed repeats covered 3,735,248 bp, corresponding to 7.96% of the genome. In *C. saccharinus*, interspersed repeats covered 6,617,796 bp, corresponding to 12.07% of the genome. Retroelements accounted for much of this difference: *C. xanthothrix* contained 1,649,099 bp of retroelements (3.51%), whereas *C. saccharinus* contained 4,424,077 bp (8.07%). DNA transposons were more similar between the two species, covering 160,597 bp in *C. xanthothrix* and 160,697 bp in *C. saccharinus*.

Detailed repeat-family summaries showed that total masked sequence reached 4,123,325 bp (8.79%) in *C. xanthothrix* (Table S7) and 6,975,358 bp (12.73%) in *C. saccharinus* (Table S8). LINEs were especially expanded in *C. saccharinus*, covering 1,710,455 bp (3.12%) compared with 209,191 bp (0.45%) in *C. xanthothrix*. LTR retrotransposons also occupied more sequence in *C. saccharinus* than in *C. xanthothrix*, with 2,653,814 bp (4.84%) in *C. saccharinus* and 1,427,245 bp (3.04%) in *C. xanthothrix*. Within LTRs, Ty1/Copia elements covered 430,822 bp in *C. xanthothrix* and 906,798 bp in *C. saccharinus*, while Gypsy/DIRS1 elements covered 818,928 bp in *C. xanthothrix* and 1,517,994 bp in *C. saccharinus*. These results indicate that the larger *C. saccharinus* genome is associated mainly with higher retroelement content rather than with changes in GC content or chromosome number.

### 3.5. Gene Prediction, Functional Annotation, and CAZyme Repertoire

Gene prediction identified 11,205 high-confidence protein-coding genes in *C. xanthothrix* and 11,881 in *C. saccharinus* (Table S11). Functional annotation rates were broadly comparable between the two species. In *C. xanthothrix*, 3645 genes (32.53%) were assigned GO terms, 4563 genes (40.72%) received KEGG KO annotations, 2862 genes (25.54%) were assigned KEGG pathway annotations, and 8325 genes (74.30%) were assigned COG annotations. In *C. saccharinus*, 3690 genes (31.06%) were assigned GO terms, 4717 genes (39.70%) received KEGG KO annotations, 2,929 genes (24.65%) were assigned KEGG pathway annotations, and 8733 genes (73.50%) were assigned COG annotations. The number of unique GO terms, KOs, pathways, and COG categories was also similar, with *C. xanthothrix* containing 10,173 unique GO terms, 3298 KOs, 750 pathways, and 24 COG categories, and *C. saccharinus* containing 10,220 unique GO terms, 3318 KOs, 752 pathways, and 24 COG categories.

CAZyme annotation showed that *C. xanthothrix* and *C. saccharinus* have comparable carbohydrate-active enzyme repertoires (Table S2). *C. xanthothrix* contained 480 predicted CAZymes, including 166 glycoside hydrolases (GH), 65 glycosyltransferases (GT), 17 polysaccharide lyases (PL), 36 carbohydrate esterases (CE), 92 auxiliary activity enzymes (AA), and 104 carbohydrate-binding modules (CBM). *C. saccharinus* contained 504 predicted CAZymes, including 181 GH, 67 GT, 17 PL, 34 CE, 99 AA, and 106 CBM. Family-level CAZyme distributions across *C. xanthothrix*, *C. saccharinus*, and related fungi are provided in Table S3, which lists 151 CAZyme families and supports more detailed comparisons of carbohydrate metabolism potential.

### 3.6. Comparative Genomics and Orthologous Group Identification

Orthogroup analysis was performed across 11 fungal genomes (Table 3 and Table S1). The comparative dataset included *C. xanthothrix*, *C. saccharinus*, *C. domesticus*, *C. radians*, *C. disseminatus*, *C. micaceus*, *Coprinopsis cinerea*, *Coprinopsis marcescibilis*, *Psathyrella aberdarensis*, *Agaricus bisporus*, and *Saccharomyces cerevisiae* as the outgroup (Table S1). Orthogroup input gene counts varied among species, ranging from 4399 genes in *S. cerevisiae* to 20,758 genes in *C. micaceus* (Table 3). The orthogroup input set contained 13,046 genes for *C. xanthothrix* and 15,802 genes for *C. saccharinus*; these values reflect the orthology-analysis input context and should not be interpreted as identical to the high-confidence gene counts reported in Table S11.

Species-specific and single-copy gene counts also varied among taxa (Table 3). *C. xanthothrix* had 45 species-specific genes and 5702 single-copy genes, while *C. saccharinus* had 109 species-specific genes and 6662 single-copy genes. Other *Coprinellus* species showed different species-specific gene counts, including 180 in *C. disseminatus*, 32 in *C. domesticus*, 1068 in *C. micaceus*, and 32 in *C. radians*. These orthology results provided the basis for single-copy phylogenetic reconstruction, divergence-time estimation, and downstream gene family expansion and contraction analysis.

### 3.7. Phylogenetic Reconstruction, Divergence Time Estimation, and Gene Family Evolution

**Figure 3:**
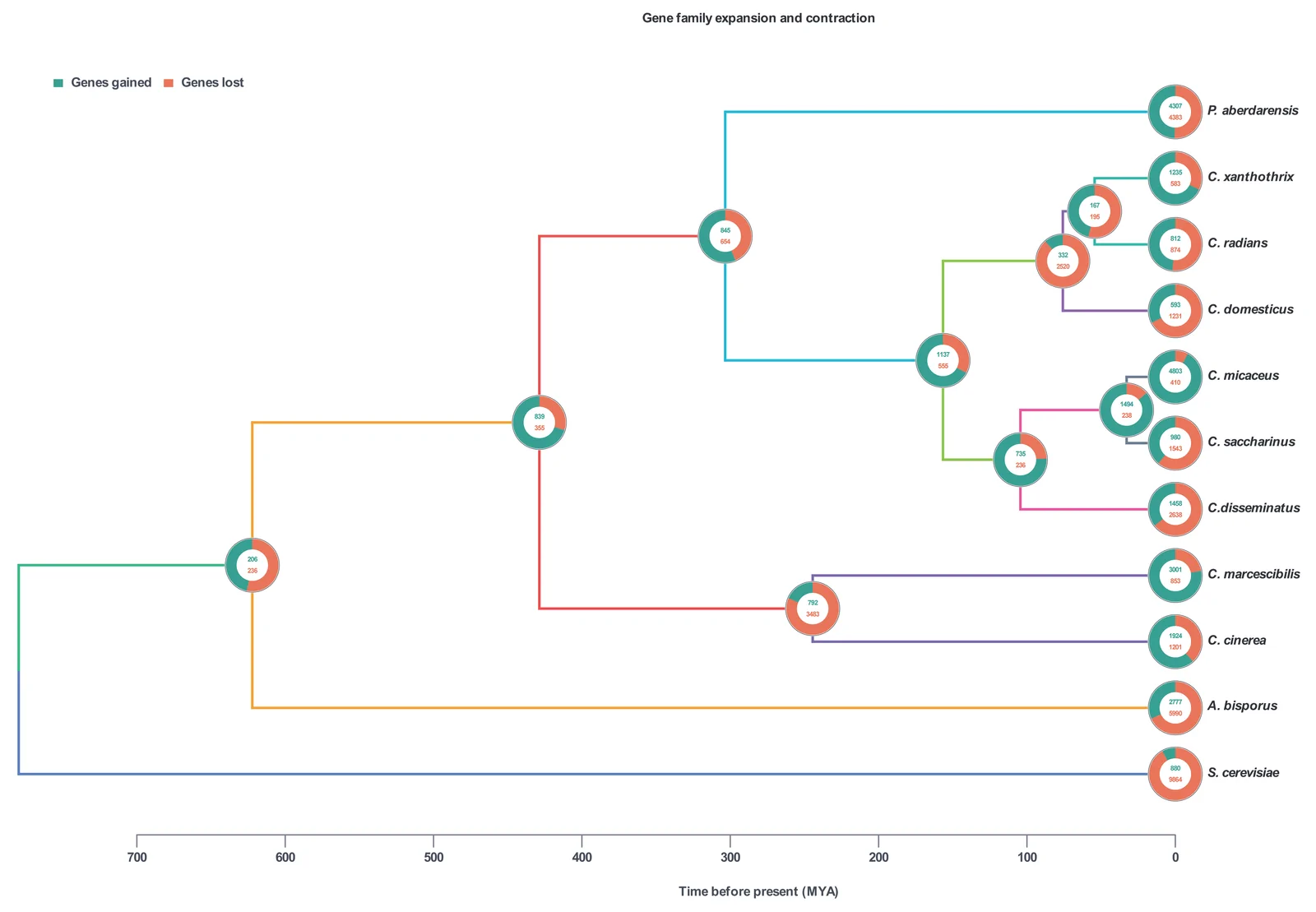
Phylogenetic placement, divergence time estimation, and gene family evolution of *C. xanthothrix* and *C. saccharinus*. A time-calibrated phylogeny of *C. xanthothrix*, *C. saccharinus*, and related fungal species was inferred from single-copy orthologues using maximum-likelihood analysis and MCMCTree, with *S. cerevisiae* used as the outgroup. Numbers at internal nodes indicate gene family expansions (green) and contractions (red) estimated using CAFE. The time scale is shown in million years ago (MYA).

Phylogenetic analysis placed *C. xanthothrix* and *C. saccharinus* in separate *Coprinellus* lineages (Figure 3). *C. xanthothrix* grouped with *C. radians*, whereas *C. saccharinus* grouped with *C. micaceus*. Divergence-time estimates showed that the split between *C. radians* and *C. xanthothrix* occurred at 54.4 Ma, with a 95% highest posterior density interval of 43.6–66.9 Ma (Table S6). The split between *C. saccharinus* and *C. micaceus* was estimated at 32.8 Ma, with a 95% interval of 20.9–54.9 Ma. Deeper nodes included the divergence between the *C. disseminatus/C. saccharinus/C. micaceus* lineage and the *C. domesticus/C. radians/C. xanthothrix* lineage at 156.6 Ma, and the divergence between *C. domesticus* and the *C. radians/C. xanthothrix* lineage at 75.8 Ma (Table S6).

CAFE analysis identified extensive gene family changes across the phylogeny (Figure 3; Table S4). On the *C. xanthothrix* terminal branch, 536 gene families were expanded, corresponding to 1235 genes gained, while 471 gene families were contracted, corresponding to 583 genes lost. This branch included 83 rapidly expanded families and 26 rapidly contracted families, for 109 total rapidly changing families. On the *C. saccharinus* terminal branch, 606 gene families were expanded, corresponding to 980 genes gained, while 1142 families were contracted, corresponding to 1543 genes lost. *C. saccharinus* showed 51 rapidly expanded families and 83 rapidly contracted families, for 134 total rapidly changing families. Among related species, *C. micaceus* showed particularly extensive expansion, with 2264 expanded families, 4803 genes gained, and 234 rapidly expanded families (Table S4).

### 3.8. GO and KEGG Enrichment of Expanded Gene Families

**Figure 4:**
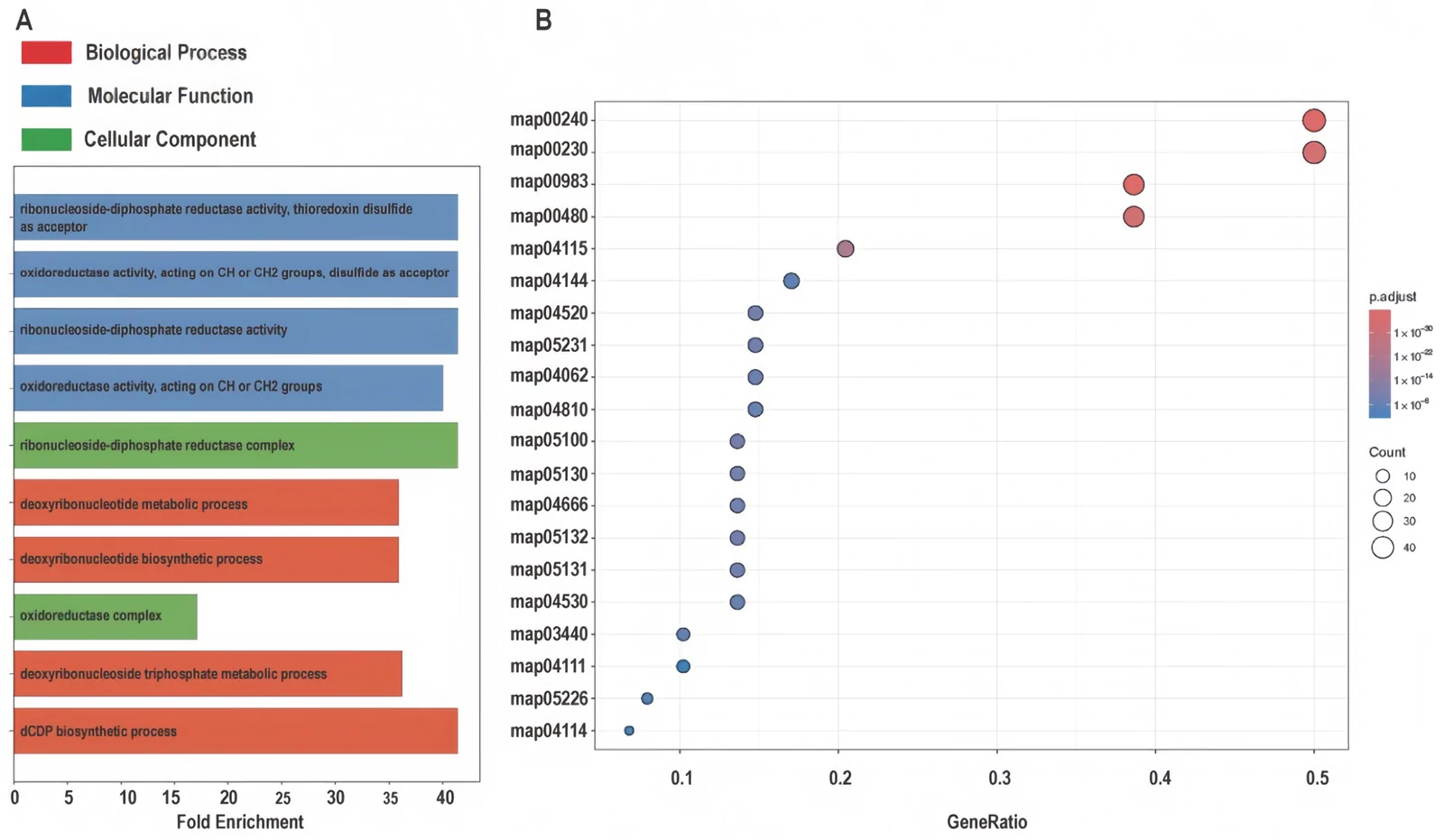
Functional enrichment analysis of expanded gene families in *C. xanthothrix*. (**A**) Gene Ontology (GO) enrichment of expanded genes. (**B**) KEGG pathway enrichment of expanded genes. The pathway names corresponding to the KEGG pathway IDs shown on the y-axis are provided in Table S9.

**Figure 5:**
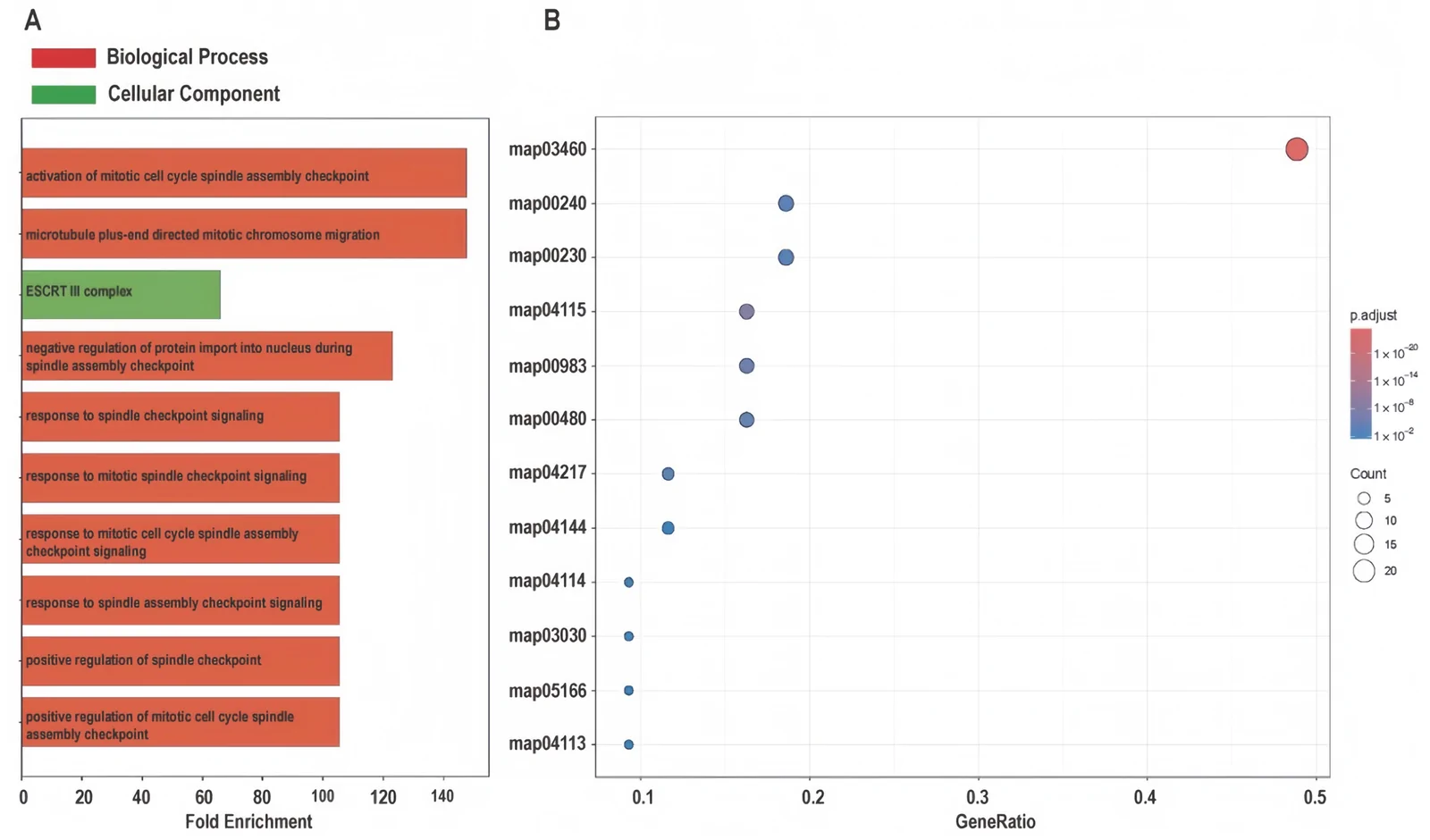
Functional enrichment analysis of expanded gene families in *C. saccharinus***.** (**A**) Gene Ontology (GO) enrichment of expanded genes. (**B**) KEGG pathway enrichment of expanded genes. The pathway names corresponding to the KEGG pathway IDs shown on the y-axis are provided in Table S9.

Expanded gene families in *C. xanthothrix* and *C. saccharinus* were enriched for functions related to nucleotide metabolism, DNA synthesis and repair, redox processes, and cellular organization (Figure 4 and Figure 5; Table S9). In *C. xanthothrix*, GO enrichment included terms associated with deoxyribonucleotide metabolism, ribonucleoside-diphosphate reductase activity, oxidoreductase activity, and other processes connected to nucleotide supply and redox balance (Figure 4A). KEGG enrichment in *C. xanthothrix* included purine metabolism (map00230), pyrimidine metabolism (map00240), glutathione metabolism (map00480), drug metabolism—other enzymes (map00983), homologous recombination (map03440), endocytosis (map04144), and regulation of actin cytoskeleton (map04810), as mapped in Table S9 (Figure 4B).

In *C. saccharinus*, GO enrichment highlighted mitotic chromosome migration, spindle checkpoint, chromosome segregation, and related cell-cycle processes (Figure 5A). KEGG enrichment included purine metabolism (map00230), pyrimidine metabolism (map00240), glutathione metabolism (map00480), DNA replication (map03030), homologous recombination (map03440), Fanconi anemia pathway (map03460), cell cycle—yeast (map04111), meiosis—yeast (map04113), endocytosis (map04144), and regulation of actin cytoskeleton (map04810), among other pathway IDs listed in Table S9 (Figure 5B). Several KEGG pathway names in Table S9 are named from animal disease or cellular systems; in this fungal context, they are interpreted as conserved molecular modules rather than literal disease phenotypes. Overall, the enrichment results support the hypothesis that lineage-specific expansions in *C. xanthothrix* and *C. saccharinus* involved nucleotide metabolism, DNA replication and repair, oxidative stress-related metabolism, and cellular proliferation or organization.

### 3.9. Genomic Synteny and Ks-Based Evolutionary Trajectory Detection

**Figure 6:**
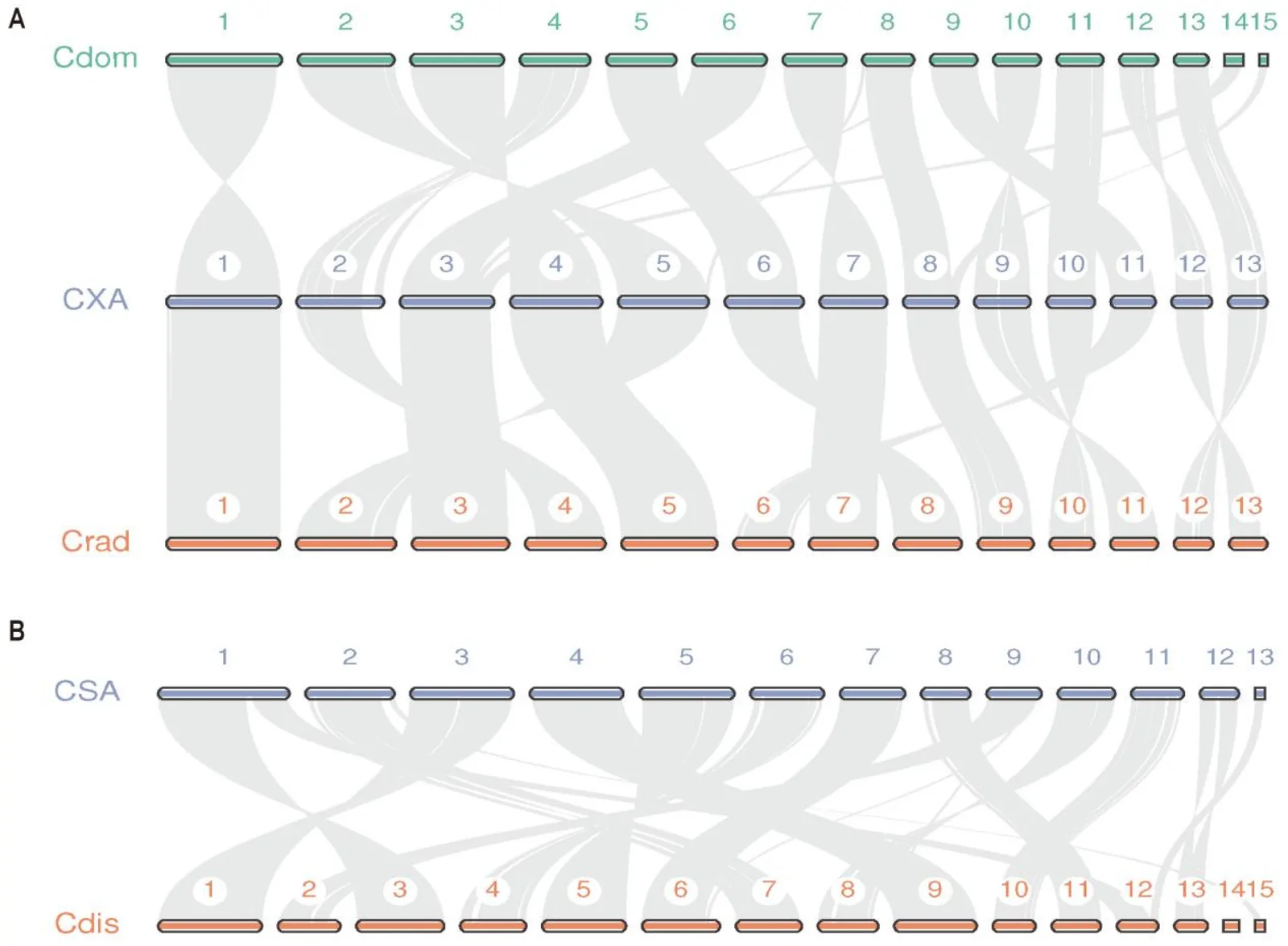
Comparative macrosynteny among *Coprinellus* species. (**A**) Chromosome-scale synteny comparison among *C. domesticus* (Cdom; 15 chromosomes), *C. xanthothrix* (CXA; 13 chromosomes), and *C. radians* (Crad; 13 chromosomes). (**B**) Chromosome-scale synteny comparison between *C. saccharinus* (CSA; 13 chromosomes) and *C. disseminatus* (Cdis; 15 chromosomes).

Chromosome-scale synteny analysis showed both conservation and rearrangement among *Coprinellus* genomes (Figure 6). For the *C. xanthothrix* comparison, *C. domesticus* contained 15 chromosomes, whereas *C. xanthothrix* and *C. radians* each contained 13 chromosomes (Figure 6A). Synteny links connected many homologous chromosomal regions across the three genomes, indicating retained macrosynteny, but cross-chromosomal links also indicated lineage-specific rearrangements. For the *C. saccharinus* comparison, *C. saccharinus* contained 13 chromosomes and *C. disseminatus* contained 15 chromosomes in the plotted comparison (Figure 6B). The synteny plot showed multiple chromosome-to-chromosome correspondences as well as rearranged segments between the two genomes. Dot-plot analyses further supported these pairwise relationships for *C. xanthothrix* versus *C. domesticus*, *C. xanthothrix* versus *C. radians*, and *C. saccharinus* versus *C. disseminatus* (Figure S3).

**Figure 7:**
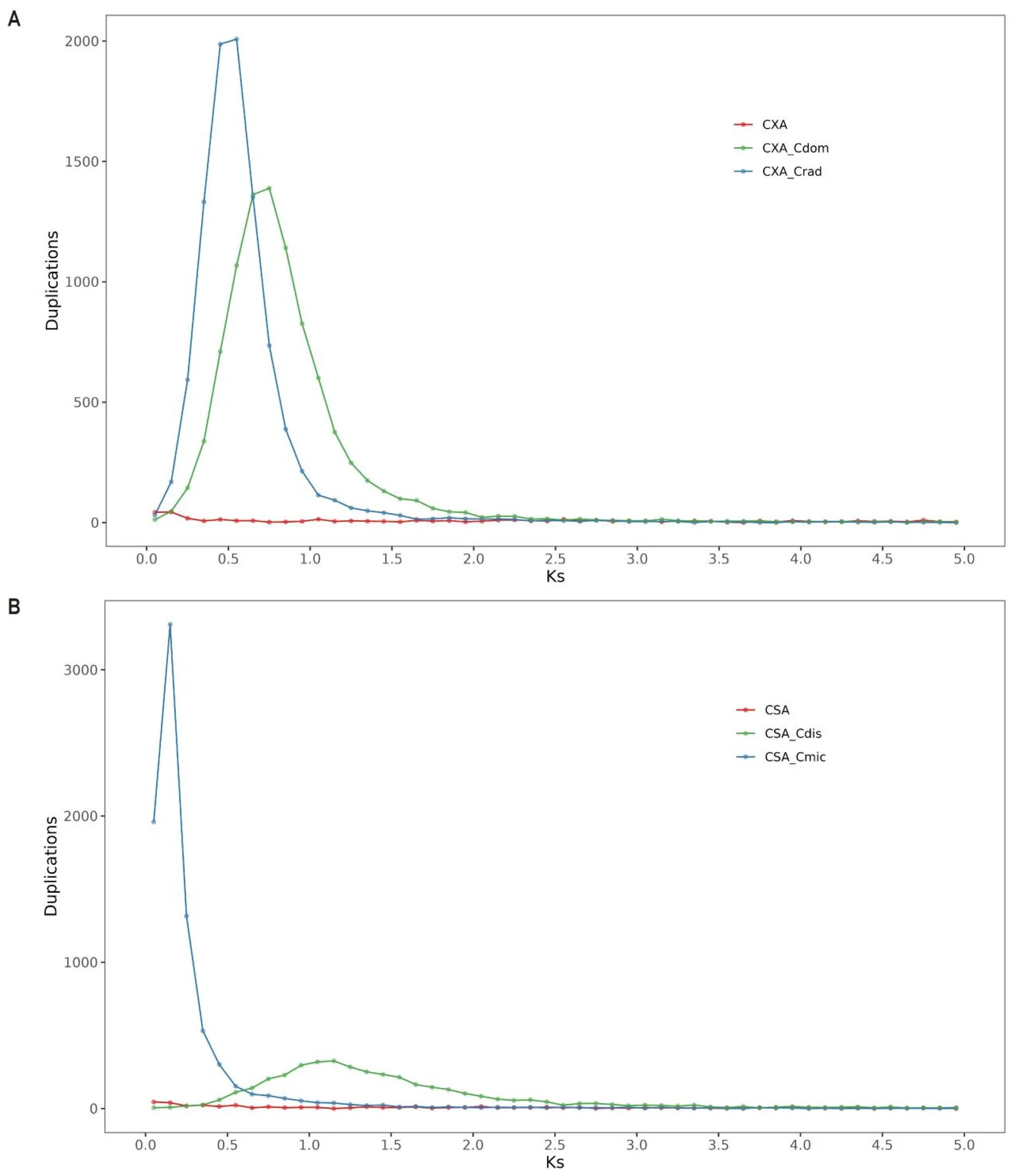
Synonymous substitution rate (Ks) distributions reveal gene duplication and divergence patterns in *Coprinellus* genomes. (**A**) Ks distributions for paralogous gene pairs within *C. xanthothrix* (CXA) and orthologous gene pairs between *C. xanthothrix* and *C. domesticus* (CXA_Cdom), and between *C. xanthothrix* and *C. radians* (CXA_Crad). (**B**) Ks distributions for paralogous gene pairs within *C. saccharinus* (CSA) and orthologous gene pairs between *C. saccharinus* and *C. disseminatus* (CSA_Cdis), and between *C. saccharinus* and *C. micaceus* (CSA_Cmic).

Ks distributions provided an additional view of duplication and divergence history (Figure 7). In *C. xanthothrix*, paralogous gene pairs showed a distribution distinct from the orthologous comparisons with *C. domesticus* and *C. radians* (Figure 7A). The ortholog distribution between *C. xanthothrix* and *C. radians* was consistent with the closer phylogenetic relationship between these species, whereas the comparison between *C. xanthothrix* and *C. domesticus* represented a more distant relationship. In *C. saccharinus*, paralogous pairs and orthologous pairs with *C. disseminatus* and *C. micaceus* showed corresponding differences in Ks distributions (Figure 7B). The comparison between *C. saccharinus* and *C. micaceus* was consistent with the close phylogenetic placement of these species. Neither *C. xanthothrix* nor *C. saccharinus* showed a dominant recent paralogous Ks peak that would support a clear recent whole-genome duplication event. Instead, the data are more consistent with lineage-specific small-scale duplication, gene family expansion, and chromosome-level rearrangement.

## 4. Discussion

The two assemblies reported here add chromosome-end-resolved genomic resources for *Coprinellus.* Both *C. xanthothrix* and *C. saccharinus* were assembled into 13 gapless chromosomes with complete telomere recovery, high BUSCO completeness, and high consensus QV. These characteristics are important because T2T genomes reduce uncertainty in subtelomeric regions and repeat-rich intervals, which are often difficult to reconstruct with fragmented assemblies [1,2,3,4,5,6]. For comparative genomics, the use of a common assembly and annotation workflow also reduces technical variation when interpreting genome size, repeat proportion, gene family dynamics, and synteny across species.

The assembly statistics support both shared architecture and species-specific divergence. Both species have 13 chromosomes and almost identical GC content, but *C. saccharinus* is 7.89 Mb larger than *C. xanthothrix*. Repeat annotation indicates that retroelement content explains a substantial fraction of this difference. *C. saccharinus* contains more than twice the retroelement sequence of *C. xanthothrix*, with especially higher LINE and LTR element coverage. Similar observations in other T2T fungal genomes have shown that complete assemblies improve the ability to quantify repetitive and chromosome-terminal sequence [3,4,5,6]. In the present data, the larger *C. saccharinus* genome therefore appears to reflect repeat accumulation rather than a broad change in gene number or GC content.

The gene annotation results show comparable functional capacity between the two species. *C. saccharinus* has more high-confidence genes than *C. xanthothrix*, but annotation rates for GO, KEGG, KEGG pathway, and COG categories are similar. CAZyme repertoires are also broadly comparable, with *C. saccharinus* carrying 504 predicted CAZymes and *C. xanthothrix* carrying 480. Because both species are saprotrophic, carbohydrate-active enzymes are expected to be relevant to organic matter decomposition. However, the class-level differences observed here are modest, and the current evidence does not support a strong functional shift in CAZyme repertoire alone. More specific ecological or substrate-use hypotheses would require transcriptomic profiling under defined substrate conditions and manual curation of family-level CAZyme functions.

Genome-enabled studies of other wood-decaying basidiomycetes provide a useful global context. Previously published genomes of *Trametes hirsuta*, *Steccherinum ochraceum*, *Pycnoporus cinnabarinus*, and multiple *Trametes* species, including *T. versicolor*, have documented extensive but lineage-variable repertoires of CAZymes, oxidoreductases, and other gene families linked to plant-biomass decomposition [58,59,60,61]. Against this background, the broadly similar class-level CAZyme complements of *C. xanthothrix* and *C. saccharinus* appear typical of saprotrophic capacity, whereas the chromosome-complete resolution of both *Coprinellus* genomes and their lineage-specific repeat and gene family patterns represent the principal novel contribution of this study. Direct numerical comparisons among studies should nevertheless be interpreted cautiously because assembly completeness, gene prediction, database versions, and CAZyme annotation pipelines differ.

The dated phylogeny places the divergence between *C. xanthothrix* and *C. radians* at 54.4 Ma and that between *C. saccharinus* and *C. micaceus* at 32.8 Ma. These estimates place the focal terminal divergences within the Paleogene, a period characterized by greenhouse climates, the Paleocene–Eocene Thermal Maximum, the Early Eocene Climatic Optimum, and subsequent long-term Cenozoic climatic reorganization [62,63]. The PETM and early Eocene hyperthermal intervals were associated with major carbon-cycle perturbations, rapid warming, vegetation turnover, range shifts, and changes in terrestrial ecosystem structure [62,63,64,65]. In Neotropical records, rapid warming across the Paleocene–Eocene boundary was associated with increased plant diversity and origination rates, suggesting that warm and reorganized ecosystems created new ecological opportunities for associated organisms [65].

Within this broader context, the Paleogene divergence of *C. xanthothrix* and *C. saccharinus* from their close relatives may have been associated with global warming, vegetation turnover, restructuring of plant-derived habitats, and ecological niche reorganization. Changes in forest composition could have altered the abundance, chemical composition, and decomposition stages of deadwood, leaf litter, and other plant residues, while simultaneously modifying microhabitat temperature, moisture, redox conditions, and competitive interactions. Under increasingly heterogeneous substrate conditions and fluctuating microenvironments, the expansion of gene families associated with nucleotide metabolism, DNA replication and repair, glutathione metabolism, and redox regulation may have contributed to nucleotide supply during hyphal growth, maintenance of genome stability, and tolerance of oxidative stress. Expansions involving endocytosis, cytoskeletal regulation, and cell-cycle processes may likewise have supported hyphal proliferation, nutrient acquisition, and colonization of newly available substrates. Together, these changes could have facilitated adaptation to heterogeneous plant residues and dynamic microhabitats, thereby contributing to lineage establishment and ecological differentiation in *C. xanthothrix* and *C. saccharinus*. This interpretation should nevertheless be regarded as a testable evolutionary hypothesis rather than a direct causal conclusion because the present study does not include fossil calibrations within *Coprinellus*, ancestral niche reconstruction, paleodistribution modelling, or experimental functional validation. Future substrate-utilization assays, stress treatments, transcriptomic analyses, and targeted gene function studies will be required to evaluate these proposed relationships.

The synteny and Ks results refine the evolutionary interpretation. Macrosynteny plots show that substantial collinearity is retained among related *Coprinellus* genomes, but the cross-chromosome ribbons indicate rearrangements after species divergence. The 13-chromosome assemblies of *C. xanthothrix* and *C. saccharinus*, compared with 15-chromosome configurations in *C. domesticus* and *C. disseminatus* in the plotted comparisons, suggest that chromosome fusion, fission, translocation, or assembly/annotation differences may contribute to the observed patterns. The current evidence supports chromosome-level reorganization, but the exact mechanisms cannot be resolved without breakpoint-level analysis and manual validation.

Ks distributions are consistent with the dated phylogeny: lower-Ks ortholog peaks occur for the closer species pairs *C. xanthothrix-C. radians* and *C. saccharinus-C. micaceus*, while more distant comparisons show broader or higher-Ks distributions. The paralogous distributions in *C. xanthothrix and C. saccharinus* do not show a dominant recent peak typical of a clear whole-genome duplication signature. Accordingly, the most conservative interpretation is that retained paralogs and segmental or small-scale duplications contribute to the observed patterns, while no strong evidence for a recent WGD event is present in either focal species.

Because this study is primarily based on genome assemblies and comparative computational analyses, the biological implications inferred from the functional enrichment analyses should be regarded as testable hypotheses. The proposed Paleogene ecological scenario is supported by divergence-time estimates and published paleoclimate evidence, while direct ecological reconstruction of these fungal lineages remains an important direction for future research. Future studies integrating transcriptomic analyses under defined substrate and stress conditions, population-level sampling, manual curation of expanded gene families, breakpoint-level synteny analyses, and functional validation of genes involved in nucleotide metabolism, DNA repair, redox regulation, and cell-cycle control will help to further test and refine these evolutionary interpretations.

## 5. Conclusions

This study generated T2T genome assemblies for *C. xanthothrix* and *C. saccharinus*, each resolved into 13 gapless chromosomes with complete telomere recovery and high assembly completeness. Comparative analyses showed that *C. saccharinus* has a larger and more repeat-rich genome than *C. xanthothrix*, largely because of its greater retroelement content, whereas the two species retain broadly comparable functional annotation profiles and CAZyme repertoires. Phylogenomic analysis grouped *C. xanthothrix* with *C. radians* and *C. saccharinus* with *C. micaceus*, with terminal divergences estimated at 54.4 and 32.8 Ma, respectively. Gene family analyses identified lineage-specific expansions enriched in nucleotide metabolism, DNA replication and repair, glutathione metabolism, redox regulation, endocytosis, cytoskeletal organization, and cell-cycle processes. Synteny and Ks analyses further revealed chromosome-level conservation and rearrangement together with lineage-specific small-scale duplication but provided no strong evidence of recent whole-genome duplication.

The Paleogene timing of the focal divergences, considered together with lineage-specific gene family expansions, suggests a possible association between long-term environmental reorganization and ecological differentiation in *C. xanthothrix* and *C. saccharinus*. This interpretation remains a testable evolutionary hypothesis rather than a causal conclusion and will require paleobiogeographic reconstruction and experimental functional validation. Collectively, the two T2T genomes provide chromosome-complete references for investigating genome evolution, ecological adaptation, and functional diversification in saprotrophic *Coprinellus* lineages.

## Figures and Tables

**Table 1:** Genome Assembly Statistics.

Feature	*C. xanthothrix*	*C. saccharinus*
Assembly level	T2T	T2T
Total genome size (Mb)	46.92	54.81
Chromosome number	13	13
Largest chromosome (Mb)	5.08 (Chr01)	7.31 (Chr01)
Smallest chromosome (Mb)	2.22 (Chr13)	0.84 (Chr13)
Gap number	0	0
GC content (%)	53.72%	53.79%
N50	4,017,987	5,059,299
N90	2,528,967	3,343,676
L50	6	5
L90	11	11
QV	38.36	39.26
Telomere motif	AACCCT	AACCCT
Telomere-capped ends	26/26 (100%)	26/26 (100%)
BUSCO (fungi_odb10)	99.20%	99.10%
NCBI/GSA Accession	PRJNA1450334/PRJCA038423	PRJNA1450334/PRJCA038423

**Table 2:** Repeat Element Statistics.

Species	Element Type	Number of Elements	Length (bp)	Percentage of Sequence (%)
*C. xanthothrix*	Retroelements	1286	1,649,099	3.51
	DNA transposons	142	160,597	0.34
	Rolling-circles	101	106,931	0.23
	Unclassified	3806	1,925,552	4.10
	Total interspersed repeats	/	3,735,248	7.96
	Small RNA	41	11,155	0.02
	Simple repeats	4609	220,747	0.47
	Low complexity	1037	58,988	0.13
*C. saccharinus*	Retroelements	2757	4,424,077	8.07
	DNA transposons	181	160,697	0.29
	Rolling-circles	13	28,761	0.05
	Unclassified	4803	2,033,022	3.71
	Total interspersed repeats	/	6,617,796	12.07
	Small RNA	127	36,128	0.07
	Simple repeats	5741	255,121	0.47
	Low complexity	1322	73,680	0.13

**Table 3:** Ortholog Statistics.

Species	Genes	Species-Specific	Single-Copy
*A. bisporus*	10,140	1546	4742
*C. saccharinus*	15,802	109	6662
*C. xanthothrix*	13,046	45	5702
*C. cinerea*	11,869	687	5422
*C.disseminatus*	13,929	180	5757
*C. domesticus*	11,784	32	5718
*C. marcescibilis*	13,294	775	5404
*C. micaceus*	20,758	1068	5977
*C. radians*	12,332	32	5713
*P. aberdarensis*	13,952	913	4614
*S. cerevisiae*	4399	659	2636

## Data Availability

The assembled genome sequences are publicly accessible through the National Center for Biotechnology Information (NCBI) under the BioProject accession PRJNA1450334. Raw sequencing reads have been archived in the Genome Sequence Archive (GSA), hosted by the China National Center for Bioinformation (CNCB), with the corresponding BioProject identifier PRJCA038423. All custom scripts developed for data analysis and figure generation are freely available via GitHub at https://github.com/huowenyanabace/T2Tfungi_custom_scripts.git (accessed on 9 March 2026).

## Supplementary Materials

The following supporting information can be downloaded at: https://www.mdpi.com/article/10.3390/jof12090678/s1, Figure S1, Per-chromosome Hi-C contact heatmaps for all 13 chromosomes of the *C. xanthothrix* T2T genome assembly; Figure S2, Per-chromosome Hi-C contact heatmaps for all 13 chromosomes of the *C. saccharinus* T2T genome assembly; Figure S3, Dot-plot analysis of pairwise whole-genome synteny between *Coprinellus* species. (A) Dot plot of *C. xanthothrix* (CXA) versus *C. domesticus* (Cdom). (B) Dot plot of *C. xanthothrix* (CXA) versus *C. radians* (Crad). (C) Dot plot of *C. saccharinus* (CSA) versus *C. disseminatus* (Cdis); Table S1, Species used for comparative genomics analysis; Table S2, Statistics of CAZyme gene numbers; Table S3, Detailed comparison of major CAZyme families; Table S4, Gene Family Evolution Summary; Table S5, Detailed Extension Statistics of *C. xanthothrix* and *C. saccharinus*; Table S6, Divergence Time Estimates with 95% HPD Intervals; Table S7, Detailed repeat element family composition of the *C. xanthothrix* T2T genomes; Table S8, Detailed repeat element family composition of the *C. saccharinus* T2T genomes; Table S9, KEGG pathway IDs and corresponding pathway names; Table S10, Sequencing Data Statistics; Table S11, Gene Annotation Statistics with MAKER.
