# Supplementary material for: Telomere-to-Telomere Genome Assemblies of Coprinellus xanthothrix and Coprinellus saccharinus Reveal Chromosome-Scale Genome Architecture and Lineage-Specific Evolutionary Dynamics
Source: J Fungi (Basel). 2026 Sep 10;12(9):678. doi: 10.3390/jof12090678 (PMC13608088; doi:10.3390/jof12090678)
Supplement: Supplementary file 1 [file jof-12-00678-s001.zip › jof-4481632-supplementary.pdf]

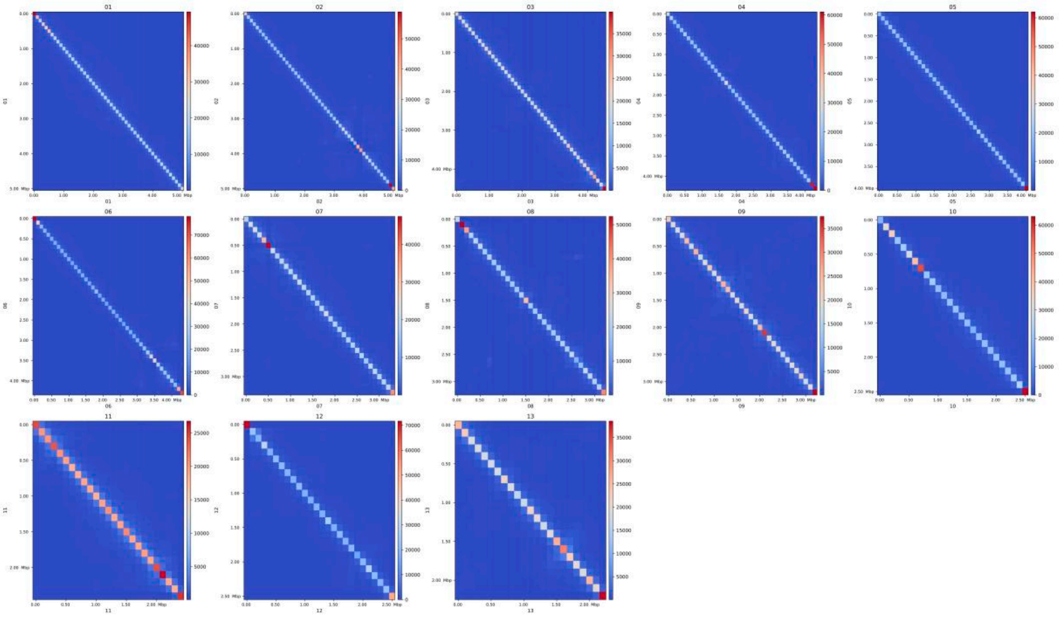

Figure S1. Per-chromosome Hi-C contact heatmaps for all 13 chromosomes of the *C. xanthothrix* T2T genome assembly.

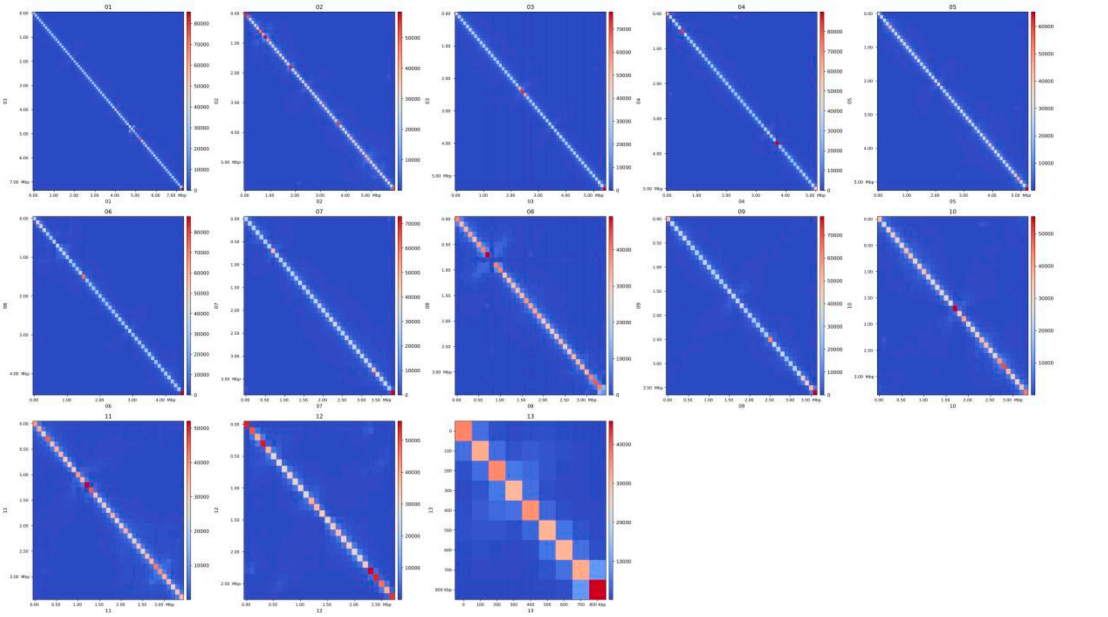

Figure S2. Per-chromosome Hi-C contact heatmaps for all 13 chromosomes of the *C. saccharinus* T2T genome assembly.

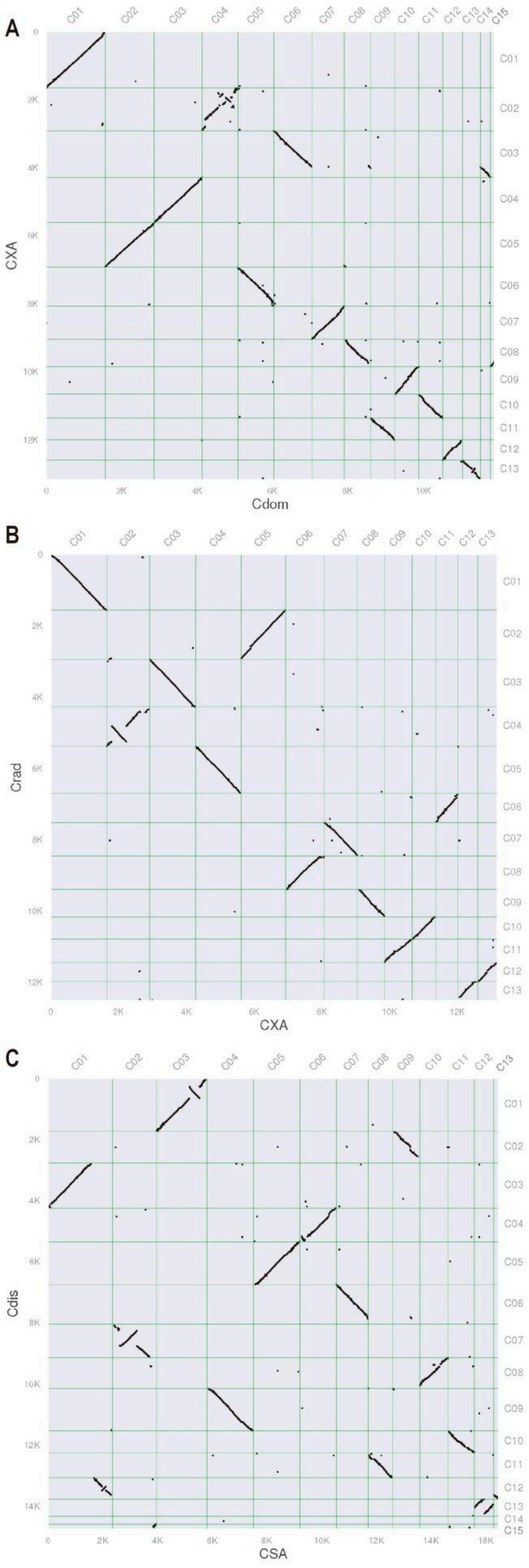

Figure S3. Dot-plot analysis of pairwise whole-genome synteny between *Coprinellus* species. (A) Dot plot of *C. xanthothrix* (CXA) versus *C. domesticus* (Cdom). (B) Dot plot of *C. xanthothrix* (CXA) versus *C. radians* (Crad). (C) Dot plot of *C. saccharinus* (CSA) versus *C. disseminatus* (Cdis).

Table S1. Species used for comparative genomics analysis

Species	Abbreviation	Lifestyle	Genome Source	Assembly accession
Coprinellus xanthothrix	CXA	Saprotrophic	This Study	GCA_059941265.1
Coprinellus saccharinus	CSA	Saprotrophic	This Study	GCA_059941275.1
Coprinellus domesticus	Cdom	Saprotrophic	NCBI	GCA_059510655.1
Coprinellus radians	Crad	Saprotrophic	NCBI	GCA_059510645.1
Coprinellus disseminatus	Cdis	Saprotrophic	NCBI	GCA_059510685.1
Coprinellus micaceus	Cmic	Saprotrophic	Ensemblgenomes	GCA_004369175
Coprinopsis cinerea	Ccin	Saprotrophic	Ensemblgenomes	GCA_000182895
Coprinopsis marcescibilis	Cmar	Saprotrophic	Ensemblgenomes	GCA_004369085.1
Psathyrella aberdarensis	Paber	Saprotrophic	Ensemblgenomes	GCA_004126415.1
Agaricus bisporus	Abis	Saprotrophic	Ensemblgenomes	GCA_000300555
Saccharomyces cerevisiae	Scere	Outgroup	Ensemblgenomes	GCA_000146045.2

Table S2. Statistics of CAZyme gene numbers

CAZyme Class	Abis	CSA	CXA	Ccin	Cdis	Cdom	Cmar	Cmic	Crad	Paber	Scere
Glycoside Hydrolases (GH)	165	181	166	172	168	169	157	203	170	174	58
Glycosyl Transferases (GT)	58	67	65	69	69	64	69	72	63	66	68
Polysaccharide Lyases (PL)	12	17	17	19	19	18	15	19	18	17	0
Carbohydrate Esterases (CE)	19	34	36	39	30	31	37	41	30	38	2
Auxiliary Activities (AA)	74	99	92	98	102	94	102	110	93	127	7
Carbohydrate-Binding Modules (CBM)	66	106	104	114	105	103	95	118	99	113	20
Total CAZymes	394	504	480	511	493	479	475	563	473	535	155

Table S3. Detailed comparison of major CAZyme families

CAZyme Family	Abis	CSA	CXA	Ccin	Cdis	Cdom	Cmar	Cmic	Crad	Paber	Scere
AA0	0	1	1	1	1	1	0	1	1	1	0
AA1	13	15	9	17	19	9	20	19	11	18	3
AA2	3	4	4	3	3	4	8	5	4	3	1
AA3	34	34	42	33	36	39	28	38	39	56	0
AA5	9	5	5	6	5	6	7	6	5	7	0
AA6	4	4	2	3	5	3	3	5	3	2	3
AA7	0	1	1	0	1	3	0	1	3	1	0
AA8	1	7	7	8	7	7	9	7	7	8	0
AA9	8	21	14	19	18	15	17	21	13	23	0
AA12	1	6	6	7	6	6	8	6	6	7	0
AA14	1	1	1	1	1	1	2	1	1	1	0
CBM0	0	2	0	0	2	1	0	1	0	0	0
CBM1	27	57	49	58	58	52	50	63	52	65	1
CBM2	5	17	16	14	17	17	10	18	14	17	0
CBM3	0	1	1	1	0	1	0	1	0	1	0
CBM4	0	1	0	2	2	0	1	0	0	0	0
CBM5	5	2	2	2	2	2	2	2	3	3	0
CBM6	4	1	2	1	2	3	0	1	2	2	0
CBM10	0	0	0	1	0	0	0	0	0	1	0
CBM12	0	0	1	0	0	0	0	0	0	0	0
CBM13	4	3	5	6	4	5	3	6	6	3	0
CBM16	0	1	1	1	1	1	1	1	1	1	0
CBM18	3	10	13	8	7	8	8	11	9	8	2

CBM19	3	1	0	1	1	0	0	1	0	0	1
CBM20	4	3	3	6	3	3	3	4	3	3	2
CBM21	0	0	0	2	0	0	2	0	1	0	4
CBM22	0	1	3	2	0	3	1	1	1	1	0
CBM24	2	1	1	1	1	0	3	1	0	2	0
CBM25	1	1	1	0	1	1	0	2	1	1	0
CBM27	0	0	0	1	0	0	2	0	0	0	0
CBM32	0	1	1	1	1	1	1	1	1	1	0
CBM35	2	1	1	1	1	1	1	2	1	1	0
CBM38	0	0	0	0	0	0	0	0	0	0	1
CBM39	0	0	0	1	0	0	1	0	0	0	0
CBM43	1	1	1	2	1	1	5	1	1	1	3
CBM48	1	1	1	1	1	1	1	1	1	1	6
CBM50	2	0	0	0	0	0	0	0	0	0	0
CBM51	2	0	0	0	0	0	0	0	0	0	0
CBM60	0	0	1	1	0	2	0	0	1	1	0
CBM61	0	0	1	0	0	0	0	0	1	0	0
CE0	0	0	0	2	0	0	2	0	0	3	0
CE1	1	3	3	4	3	3	4	3	3	7	0
CE2	0	0	0	1	0	0	1	0	0	0	0
CE3	0	5	1	1	3	2	3	10	1	2	0
CE4	6	14	17	13	11	11	13	14	12	10	2
CE5	5	1	0	6	1	0	0	3	0	1	0
CE8	1	0	0	0	0	0	0	0	0	0	0
CE9	1	1	1	1	1	1	1	1	1	1	0
CE12	2	1	1	1	1	1	1	1	1	1	0

CE15	1	7	10	7	7	8	9	7	8	10	0
CE16	1	1	2	2	2	4	2	1	3	2	0
CE17	1	1	1	1	1	1	1	1	1	1	0
GH0	3	4	3	3	1	6	2	3	4	5	5
GH1	2	3	3	2	3	2	2	3	3	2	0
GH2	2	2	2	2	2	2	2	3	2	2	0
GH3	8	7	6	7	7	6	5	8	6	6	0
GH5	18	22	24	24	21	22	27	27	24	32	5
GH6	1	2	2	5	2	3	3	2	3	5	0
GH7	1	6	2	6	5	5	4	7	5	5	0
GH9	1	1	1	1	1	1	1	1	1	1	0
GH10	2	6	7	5	7	7	4	7	8	6	0
GH11	2	6	4	4	3	4	2	5	2	5	0
GH12	2	0	0	0	0	0	0	0	0	0	0
GH13	7	7	7	9	7	7	8	8	7	6	9
GH15	2	2	2	4	2	2	2	3	2	2	1
GH16	20	29	27	26	26	25	23	36	27	21	5
GH17	2	2	3	2	3	3	2	3	3	2	4
GH18	12	9	9	9	8	9	9	8	8	10	2
GH20	4	2	2	2	2	2	2	2	2	3	0
GH25	4	2	0	0	1	0	0	4	0	0	0
GH27	4	1	1	0	1	1	0	1	0	1	0
GH28	3	3	3	2	4	2	2	5	3	2	1
GH29	1	0	0	0	0	0	0	0	0	0	0
GH30	3	3	2	3	3	3	3	5	4	2	0
GH31	6	2	2	3	2	2	2	2	2	2	1

GH32	0	0	0	0	0	0	0	0	0	0	1
GH35	1	2	2	0	2	1	1	4	2	2	0
GH37	2	2	2	2	2	2	2	2	2	3	2
GH38	1	1	1	1	1	1	1	1	1	1	1
GH43	4	4	6	4	5	7	3	4	6	2	0
GH44	1	1	1	1	1	1	1	1	1	0	0
GH47	6	8	7	8	7	7	6	6	7	7	3
GH51	1	0	1	1	1	1	1	1	1	1	0
GH53	1	1	1	1	1	1	1	1	1	1	0
GH55	1	3	3	1	3	3	2	3	3	3	0
GH62	0	2	2	3	2	2	2	2	1	3	0
GH63	2	2	1	2	2	1	2	2	1	1	2
GH65	0	0	0	0	0	0	0	0	0	0	1
GH71	2	3	3	4	3	3	4	3	3	4	0
GH72	1	1	1	2	1	1	5	1	1	1	5
GH74	1	1	1	1	1	1	1	1	1	1	0
GH76	1	0	1	0	0	1	0	0	1	0	2
GH78	4	0	0	0	0	0	0	0	0	0	0
GH79	4	7	5	3	7	5	2	9	5	6	0
GH81	0	0	0	0	0	0	0	0	0	0	2
GH85	1	1	1	2	1	1	2	1	1	1	0
GH88	1	1	1	1	1	1	1	1	1	0	0
GH92	5	3	3	3	3	3	2	2	2	3	0
GH95	1	0	0	0	0	0	0	0	0	0	0
GH105	3	2	0	2	0	1	1	1	1	1	0
GH115	2	2	1	1	1	1	1	1	1	3	0

GH125	1	1	1	1	1	1	1	1	1	1	0
GH128	0	2	1	2	2	1	2	1	2	1	0
GH131	1	1	1	1	1	1	1	1	1	1	0
GH132	0	0	0	0	0	0	0	0	0	0	5
GH133	1	1	1	1	1	1	1	1	1	1	1
GH145	1	1	1	1	1	1	1	1	1	1	0
GH152	3	6	4	3	5	4	4	6	4	4	0
GH154	1	1	1	1	1	1	1	2	1	1	0
GH162	1	0	0	0	0	0	0	0	0	0	0
GT0	2	3	3	3	3	3	3	3	3	3	0
GT1	2	1	1	3	1	1	2	1	1	1	3
GT2	13	19	16	15	16	16	16	19	16	19	5
GT3	1	1	1	1	1	1	1	1	1	1	2
GT4	4	4	4	4	4	4	4	4	4	2	3
GT5	1	1	1	1	1	1	1	1	1	1	0
GT8	1	2	2	2	2	2	1	3	1	2	3
GT15	3	4	2	5	4	2	8	4	3	3	9
GT17	1	1	1	0	1	1	0	1	1	2	0
GT20	3	3	3	3	3	3	3	3	3	3	4
GT21	1	1	1	1	1	1	1	1	1	1	0
GT22	3	3	3	4	3	3	4	3	3	3	4
GT24	1	1	1	1	1	1	1	1	1	1	1
GT31	1	1	1	1	1	1	1	1	1	1	0
GT32	1	1	1	1	1	1	1	1	1	1	4
GT33	1	1	1	1	1	1	1	1	1	2	1
GT34	0	0	0	0	0	0	0	0	0	0	2

GT35	1	2	1	1	1	2	1	2	1	1	1
GT39	3	3	3	3	3	3	3	3	3	3	7
GT48	2	2	4	2	6	2	2	2	2	2	3
GT49	1	1	1	1	1	1	0	1	1	1	0
GT50	1	1	1	1	1	1	1	1	1	1	1
GT57	2	2	2	2	2	2	2	2	2	2	2
GT58	1	1	1	1	1	1	1	1	1	1	1
GT59	1	1	1	1	1	1	1	1	1	0	1
GT62	0	0	0	0	0	0	0	0	0	0	3
GT66	1	1	1	1	1	1	1	3	1	1	1
GT69	2	2	2	3	2	2	2	2	2	3	0
GT71	0	0	0	0	0	0	0	0	0	0	6
GT76	0	0	0	0	0	0	0	0	0	0	1
GT90	4	4	6	7	6	6	7	6	6	5	0
PL1	2	2	1	1	2	1	0	1	1	1	0
PL3	1	0	1	2	1	1	2	1	1	2	0
PL4	1	3	2	2	2	2	1	2	2	2	0
PL8	1	2	2	1	2	2	1	3	2	1	0
PL14	3	6	6	7	7	7	6	7	7	5	0
PL26	1	0	0	0	0	0	0	0	0	0	0
PL35	1	2	2	3	2	2	2	3	2	4	0
PL38	1	1	2	2	2	2	2	1	2	1	0
PL42	1	1	1	1	1	1	1	1	1	1	0

Table S4. Gene Family Evolution Summary

Nodes	Expansions	Genes Gained	Equal	Contractions	Genes Lost	Families Lost	Average Expansion	Rapid Expansions	Rapid Contractions	Total Rapid Changes
<11>	684	1494	10431	210	238	130	0.110905	91	4	95
Paber<20>	1219	4307	5894	4212	4383	3772	-0.00671082	70	3	73
Cdom<14>	377	593	10062	886	1231	466	-0.0563355	20	63	83
Cmar<6>	831	3001	9844	650	853	451	0.189669	62	10	72
CXA<18>	536	1235	10318	471	583	245	0.0575717	83	26	109
<3>	140	206	10959	226	236	215	-0.00264901	0	0	0
Scere<0>	578	880	2218	8529	9864	7995	-0.793289	0	7	7
Cdis<8>	727	1458	8211	2387	2638	2045	-0.104194	53	20	73
<5>	456	792	7468	3401	3483	3254	-0.237616	17	1	18
Ccin<4>	747	1924	9823	755	1201	485	0.0638411	30	21	51
<17>	129	167	11032	164	195	91	-0.00247241	6	10	16
<15>	253	332	8692	2380	2520	2154	-0.193201	10	10	20
<13>	586	1137	10321	418	555	308	0.0513907	45	6	51
Abis<2>	692	2777	5036	5597	5990	5126	-0.283709	13	1	14
<19>	410	845	10276	639	654	600	0.0168653	36	0	36
<7>	455	839	10594	276	355	246	0.0427373	10	2	12
<9>	454	735	10640	231	236	198	0.0440618	33	1	34
CSA<10>	606	980	9577	1142	1543	727	-0.049713	51	83	134
Cmic<12>	2264	4803	8725	336	410	217	0.387903	234	15	249
Crad<16>	438	812	10273	614	874	303	-0.00547461	47	62	109

Note: Nodes with only numbers (e.g., <11>, <13>) represent ancestral internal nodes in the phylogenetic tree. CAFE labeled tree:

(Scere<0>,(Abis<2>,((Ccin<4>,Cmar<6>)<5>,(((Cdis<8>,(CSA<10>,Cmic<12>)<11>)<9>,(Cdom<14>,(Crad<16>,CXA<18>)<17>)<15>)<13>,Paber<20>)<19>)<7>)<3>)<1>

Table S5. Detailed Extension Statistics of *C. xanthothrix* and *C. saccharinus*

Species	Chromosome	Terminus	Extension Length (bp)	Motif	Repeat Count
C. *xanthothrix*	Chr01	3prime	155584	AGGGTT	15
C. *xanthothrix*	Chr01	5prime	34865	AACCCT	16
C. *xanthothrix*	Chr02	3prime	1475	AGGGTT	14
C. *xanthothrix*	Chr02	5prime	91	AACCCT	14
C. *xanthothrix*	Chr03	3prime	2330	AGGGTT	20
C. *xanthothrix*	Chr03	5prime	87	AACCCT	14
C. *xanthothrix*	Chr04	3prime	85	AGGGTT	14
C. *xanthothrix*	Chr04	5prime	98	AACCCT	16
C. *xanthothrix*	Chr05	3prime	14471	AGGGTT	16
C. *xanthothrix*	Chr05	5prime	89	AACCCT	14
C. *xanthothrix*	Chr06	3prime	175350	AGGGTT	14
C. *xanthothrix*	Chr06	5prime	44612	AACCCT	13
C. *xanthothrix*	Chr07	3prime	92	AGGGTT	15
C. *xanthothrix*	Chr07	5prime	103	AACCCT	12
C. *xanthothrix*	Chr08	3prime	225	AGGGTT	15
C. *xanthothrix*	Chr08	5prime	35562	AACCCT	16
C. *xanthothrix*	Chr09	3prime	20841	AGGGTT	14
C. *xanthothrix*	Chr09	5prime	107506	AACCCT	17
C. *xanthothrix*	Chr10	3prime	118088	AGGGTT	17
C. *xanthothrix*	Chr10	5prime	98	AACCCT	15
C. *xanthothrix*	Chr11	3prime	16262	AGGGTT	19
C. *xanthothrix*	Chr11	5prime	18037	AACCCT	19
C. *xanthothrix*	Chr12	3prime	82620	AGGGTT	15
C. *xanthothrix*	Chr12	5prime	139914	AACCCT	14
C. *xanthothrix*	Chr13	3prime	148	AGGGTT	18
C. *xanthothrix*	Chr13	5prime	36364	AACCCT	18
C.saccharinus	Chr01	3prime	153876	AGGGTT	22
C.saccharinus	Chr01	5prime	921	AACCCT	34
C.saccharinus	Chr02	3prime	67792	AGGGTT	27
C.saccharinus	Chr02	5prime	23253	AACCCT	28
C.saccharinus	Chr03	3prime	6298	AGGGTT	29
C.saccharinus	Chr03	5prime	22212	AACCCT	25
C.saccharinus	Chr04	3prime	195	AGGGTT	32
C.saccharinus	Chr04	5prime	28266	AACCCT	28
C.saccharinus	Chr05	3prime	55228	AGGGTT	28
C.saccharinus	Chr05	5prime	177968	AACCCT	24
C.saccharinus	Chr06	3prime	15061	AGGGTT	30
C.saccharinus	Chr06	5prime	68452	AACCCT	28
C.saccharinus	Chr07	3prime	171	AGGGTT	28

C.saccharinus	Chr07	5prime	26186	AACCCT	32
C.saccharinus	Chr08	3prime	24326	AGGGTT	26
C.saccharinus	Chr08	5prime	3336	AACCCT	32
C.saccharinus	Chr09	3prime	150546	AGGGTT	32
C.saccharinus	Chr09	5prime	132100	AACCCT	30
C.saccharinus	Chr10	3prime	8286	AGGGTT	30
C.saccharinus	Chr10	5prime	191	AACCCT	27
C.saccharinus	Chr11	3prime	140511	AGGGTT	29
C.saccharinus	Chr11	5prime	5947	AACCCT	25
C.saccharinus	Chr12	3prime	63149	AGGGTT	26
C.saccharinus	Chr12	5prime	87860	AACCCT	22
C.saccharinus	Chr13	3prime	6782	AGGGTT	29
C.saccharinus	Chr13	5prime	37040	AACCCT	25

Table S6. Divergence Time Estimates with 95% HPD Intervals

Node	Comparison	Median Age (MYA)	95% HPD Lower (MYA)	95% HPD Upper (MYA)
Root (Node 1)	Scere vs all remaining taxa	779.6	686.7	860.4
Node 3	Abis vs Ccin + Cmar + Cdis + CSA + Cmic + Cdom + Crad + CXA + Paber	622.2	573.3	698.4
Node 7	Ccin + Cmar vs Cdis + CSA + Cmic + Cdom + Crad + CXA + Paber	428.7	357.6	519.3
Node 19	Cdis + CSA + Cmic + Cdom + Crad + CXA vs Paber	303.4	246.2	369.3
Node 5	Ccin vs Cmar	244.4	124.7	324.2
Node 13	Cdis + CSA + Cmic vs Cdom + Crad + CXA	156.6	125.8	198.0
Node 9	Cdis vs CSA + Cmic	104.4	79.6	139.7
Node 15	Cdom vs Crad + CXA	75.8	61.4	91.9
Node 17	Crad vs CXA	54.4	43.6	66.9
Node 11	CSA vs Cmic	32.8	20.9	54.9

Table S7. Detailed repeat element family composition of the *C. xanthothrix* T2T genomes

Category	Subcategory	Family	Number of Elements	Length (bp)	Percentage of Sequence (%)
Retroelements	—	Total Retroelements	1286	1649099	3.51
Retroelements	SINEs	Total SINEs	54	12663	0.03
Retroelements	Penelope	Total Penelope	0	0	0
Retroelements	LINEs	Total LINEs	110	209191	0.45
Retroelements	LINEs	CRE/SLACS	0	0	0
Retroelements	LINEs	L2/CR1/Rex	0	0	0
Retroelements	LINEs	R1/LOA/Jockey	0	0	0
Retroelements	LINEs	R2/R4/NeSL	0	0	0
Retroelements	LINEs	RTE/Bov-B	28	3981	0.01
Retroelements	LINEs	L1/CIN4	0	0	0
Retroelements	LTR elements	Total LTR elements	1122	1427245	3.04
Retroelements	LTR elements	BEL/Pao	0	0	0
Retroelements	LTR elements	Ty1/Copia	382	430822	0.92
Retroelements	LTR elements	Gypsy/DIRS1	441	818928	1.75
Retroelements	LTR elements	Retroviral	0	0	0
DNA transposons	—	Total DNA transposons	142	160597	0.34
DNA transposons	—	hobo-Activator	0	0	0
DNA transposons	—	Tc1-IS630-Pogo	0	0	0
DNA transposons	—	En-Spm	0	0	0
DNA transposons	—	MULE-MuDR	0	0	0
DNA transposons	—	PiggyBac	21	3128	0.01
DNA transposons	—	Tourist/Harbinger	7	15008	0.03
DNA transposons	—	Other (Mirage, P-element, Transib)	0	0	0
Rolling-circles	—	Rolling-circles	101	106931	0.23
Unclassified	—	Unclassified	3806	1925552	4.1
Total interspersed repeats	—	Total interspersed repeats	—	3735248	7.96
Small RNA	—	Small RNA	41	11155	0.02
Satellites	—	Satellites	0	0	0
Simple repeats	—	Simple repeats	4609	220747	0.47
Low complexity	—	Low complexity	1037	58988	0.13
Total masked	—	Total masked	—	4123325	8.79

Table S8. Detailed repeat element family composition of the *C. saccharinus* T2T genomes

Category	Subcategory	Family	Number of Elements	Length (bp)	Percentage of Sequence (%)
Retroelements	—	Total Retroelements	2757	4424077	8.07
Retroelements	SINEs	Total SINEs	127	36128	0.07
Retroelements	Penelope	Total Penelope	13	23680	0.04
Retroelements	LINEs	Total LINEs	726	1710455	3.12
Retroelements	LINEs	CRE/SLACS	0	0	0
Retroelements	LINEs	L2/CR1/Rex	0	0	0
Retroelements	LINEs	R1/LOA/Jockey	0	0	0
Retroelements	LINEs	R2/R4/NeSL	0	0	0
Retroelements	LINEs	RTE/Bov-B	28	2632	0
Retroelements	LINEs	L1/CIN4	0	0	0
Retroelements	LTR elements	Total LTR elements	1891	2653814	4.84
Retroelements	LTR elements	BEL/Pao	16	5612	0.01
Retroelements	LTR elements	Ty1/Copia	627	906798	1.65
Retroelements	LTR elements	Gypsy/DIRS1	770	1517994	2.77
Retroelements	LTR elements	Retroviral	0	0	0
DNA transposons	—	Total DNA transposons	181	160697	0.29
DNA transposons	—	hobo-Activator	0	0	0
DNA transposons	—	Tc1-IS630-Pogo	9	10434	0.02
DNA transposons	—	En-Spm	0	0	0
DNA transposons	—	MULE-MuDR	24	34998	0.06
DNA transposons	—	PiggyBac	0	0	0
DNA transposons	—	Tourist/Harbinger	0	0	0
DNA transposons	—	Other (Mirage, P-element, Transib)	0	0	0
Rolling-circles	—	Rolling-circles	13	28761	0.05
Unclassified	—	Unclassified	4803	2033022	3.71
Total interspersed repeats	—	Total interspersed repeats	—	6617796	12.07
Small RNA	—	Small RNA	127	36128	0.07
Satellites	—	Satellites	0	0	0
Simple repeats	—	Simple repeats	5741	255121	0.47
Low complexity	—	Low complexity	1322	73680	0.13
Total masked	—	Total masked	—	6975358	12.73

Table S9. KEGG pathway IDs and corresponding pathway names.

Pathway ID	Pathway name
map00230	Purine metabolism
map00240	Pyrimidine metabolism
map00480	Glutathione metabolism
map00983	Drug metabolism - other enzymes
map03030	DNA replication
map03440	Homologous recombination
map03460	Fanconi anemia pathway
map04062	Chemokine signaling pathway
map04111	Cell cycle - yeast
map04113	Meiosis - yeast
map04114	Oocyte meiosis
map04115	p53 signaling pathway
map04144	Endocytosis
map04217	Necroptosis
map04520	Adherens junction
map04530	Tight junction
map04666	Fc gamma R-mediated phagosome formation
map04810	Regulation of actin cytoskeleton
map05100	Bacterial invasion of epithelial cells
map05130	Pathogenic Escherichia coli infection
map05131	Shigellosis
map05132	Salmonella infection
map05166	Human T-cell leukemia virus 1 infection
map05226	Gastric cancer
map05231	Choline metabolism in cancer

Table S10. Sequencing Data Statistics.

Species	Data Type	TotalBase of Raw Data (G)	TotalBase of Clean Data (G)	TotalReads of Raw Data (M)	TotalReads of Clean Data (M)	Coverage (X)
C. xanthothrix	Short-read WGS	15.22	15.12	101.48	101.47	324X
	Long-read	10.76	10.38	1.77	1.62	228X
	Hi-C	10.85	10.84	36.18	36.18	231X
	RNA-seq	5.49	/	0.11	/	/
C. saccharinus	Short-read WGS	10.28	10.03	68.52	67.92	187X
	Long-read	12.57	11.96	0.54	0.50	229X
	Hi-C	19.11	19.09	63.69	63.69	349X
	RNA-seq	9.12	/	0.19	/	/

Table S11. Gene Annotation Statistics with MAKER.

Species	Category	Count	Percentage (%)
C. xanthothrix	Total Genes	11205	100.00
	GO Annotated	3645	32.53
	KEGG KO Annotated	4563	40.72
	KEGG Pathway Annotated	2862	25.54
	COG Annotated	8325	74.30
	Unique GO Terms	10173	/
	Unique KEGG KOs	3298	/
	Unique KEGG Pathways	750	/
	Unique COG Categories	24	/
C. saccharinus	Total Genes	11881	100.00
	GO Annotated	3690	31.06
	KEGG KO Annotated	4717	39.70
	KEGG Pathway Annotated	2929	24.65
	COG Annotated	8733	73.50
	Unique GO Terms	10220	/
	Unique KEGG KOs	3318	/
	Unique KEGG Pathways	752	/
	Unique COG Categories	24	/
